# Multi-stage malaria parasite recognition by deep learning

**DOI:** 10.1093/gigascience/giab040

**Published:** 2021-06-17

**Authors:** Sen Li, Zeyu Du, Xiangjie Meng, Yang Zhang

**Affiliations:** College of Science, Harbin Institute of Technology, Taoyuan Street, Nanshan District, Shenzhen, Guangdong 518055, China; College of Science, Harbin Institute of Technology, Taoyuan Street, Nanshan District, Shenzhen, Guangdong 518055, China; College of Science, Harbin Institute of Technology, Taoyuan Street, Nanshan District, Shenzhen, Guangdong 518055, China; College of Science, Harbin Institute of Technology, Taoyuan Street, Nanshan District, Shenzhen, Guangdong 518055, China

**Keywords:** malaria, multi-stage recognition, microscopic image analysis, knowledge transfer, graph convolutional network, deep learning

## Abstract

**Motivation:**

Malaria, a mosquito-borne infectious disease affecting humans and other animals, is widespread in tropical and subtropical regions. Microscopy is the most common method for diagnosing the malaria parasite from stained blood smear samples. However, this technique is time consuming and must be performed by a well-trained professional, yet it remains prone to errors. Distinguishing the multiple growth stages of parasites remains an especially challenging task.

**Results:**

In this article, we develop a novel deep learning approach for the recognition of malaria parasites of various stages in blood smear images using a deep transfer graph convolutional network (DTGCN). To our knowledge, this is the first application of graph convolutional network (GCN) on multi-stage malaria parasite recognition in such images. The proposed DTGCN model is based on unsupervised learning by transferring knowledge learnt from source images that contain the discriminative morphology characteristics of multi-stage malaria parasites. This transferred information guarantees the effectiveness of the target parasite recognition. This approach first learns the identical representations from the source to establish topological correlations between source class groups and the unlabelled target samples. At this stage, the GCN is implemented to extract graph feature representations for multi-stage malaria parasite recognition. The proposed method showed higher accuracy and effectiveness in publicly available microscopic images of multi-stage malaria parasites compared to a wide range of state-of-the-art approaches. Furthermore, this method is also evaluated on a large-scale dataset of unseen malaria parasites and the *Babesia* dataset.

**Availability:**

Code and dataset are available at https://github.com/senli2018/DTGCN_2021 under a MIT license.

## Introduction

The main source of malaria is the parasites from the Plasmodium group, which are transmitted to people though the bites of infected mosquitoes. People with malaria experience fever, chill, and a flu-like illness [1]. According to the World Health Organization, there were 228 million cases of malaria worldwide, which resulted in ∼405,000 deaths, in 2018. Approximately 94% of these deaths occurred in the African region [2]. Moreover, the risk of infectious disease (especially malaria) transmission was probably increased because severe flooding inundated many regions in Asia in 2020 [3]. This can make malaria, one of the most serious public health problems, spread worldwide. This urgent situation prompted new malaria research, and the reported results showed that the hazard of malaria illnesses and deaths can be significantly reduced by accurate and affordable diagnostic testing, enabling better disease monitoring and control interventions.

Malaria is usually diagnosed by the microscopic examination of blood films, and hundreds of millions of blood films are examined every year for malaria diagnosis [2, 4]. Although this is the most commonly used technique, the process of examining films under the microscope is tedious and susceptible to error. Therefore, a considerable number of studies on computer-aided malaria detection systems have been proposed [4, 5]. For example, classification methods have been applied to discriminate between infected and uninfected RBCs in thin smears or to identify parasites in thick smears, ranging from decision trees to basic artificial neural networks [6]. Furthermore, recent studies have proven that malaria diagnosis based on deep learning architecture can significantly outperform models based on conventional classifiers [7–10]. Liang et al. [7] applied a convolutional neural network (CNN) approach to discriminate between infected and uninfected cells in thin blood smears, resulting in 97.37% accuracy on 27,578 single-cell images. In addition, Dong et al. [8] evaluated 3 types of well-known CNNs, including LeNet, AlexNet, and GoogLeNet, which all achieved classification accuracies of >95%. Gopakumar et al. [9] used CNN operating on a focus stack for automated quantitative detection of malaria parasites from blood smear samples with improved sensitivity (97.06%) and specificity (98.50%). Hung and Carpenter [10] further developed a faster region-based CNN approach for object segmentation on malaria parasite images. The superior experimental results on the segmentation of 40,612 images demonstrate the effectiveness of the proposed model over the state-of-the-art method of traditional segmentation plus machine learning. This model also classified all objects for the segmented cells, finding that learning to distinguish features between infected classes is very challenging: only 59% accuracy was achieved. Thus, it falls to binary classification to identify the objects as RBCs or not, with an accuracy of 98%. Narayanan et al. [26] proposed a fast CNN architecture and compared it with AlexNet, ResNet, VGG-16, and DenseNet models for malaria detection. Findings showed that all tested methods achieved >96% accuracies. Similarly, Narayanan et al. [27] investigated the detection of malaria by using deep neural networks (GoogLeNet and ResNet), and obtained accuracies >96%.

The aforementioned methods are particularly useful in detecting a single stage of the malaria parasite, normally the ring form. However, the life cycle of the malaria parasites is complicated. The entire cycle involves multiple morphological changes in human blood. As illustrated in Supplementary Fig. S1a, malaria parasites develop multi-stage forms with distinct microscopic presentations during the intraerythrocytic cycle, including gametocytes, rings, trophozoites, and schizonts [11]. Until now, an accurate multi-stage malaria detection system has not been devised because of the morphological differences across multi-stage parasites and variations in images captured from different technicians, laboratories, clinics, and regions. Additionally, colour variation, resulting from differences in staining pH, time, purity of dye, duration of the staining procedure, and sensor settings (Supplementary Fig. S1b), is another challenge of multi-stage detection. All those morphological and hardware variations degrade the performance of the previously developed models. Therefore, simple adaptation of existing single-stage classification models will lead to poor performance in multi-stage malaria parasite recognition. Another important problem is the lack of multi-stage parasite training images with a balanced class distribution because of the dominance of ring-stage parasites and red blood cells (RBCs) captured under microscope.

To overcome the challenges in both variations and data imbalance for multi-stage malaria parasite recognition, we use a transfer learning strategy that takes advantage of the prior knowledge from the labelled source domain (existing scenario) to train the recognition model and apply it to an unlabelled target domain (even unseen scenario) for detection. On the other hand, the problem of data imbalance can be addressed by implementing graph convolutional network (GCN) on the established topological correlations between source class groups and target features to bridge the different class distribution gaps. Specifically, GCNs have been proposed whereby node features, aggregated from adjacent neighbours and different nodes, can share the same transfer function. Thus, the aggregated nodes can exploit more discriminative information according to the topological graph structure of the node features than directly utilizing CNN on a single image.

In this context, DTGCN is proposed for multi-stage malaria parasite recognition and classification, which consists of a CNN-based feature extractor, a source transfer graph building component, and an unsupervised GCN. A thorough review of the literature reveals that none of the previously reported studies have attempted to explore the advantages of deep learning for multi-stage malaria parasite recognition. To demonstrate the effectiveness of the proposed DTGCN, we conducted experiments on 2 public malaria parasite image datasets, which are available from the Broad Bioimage Benchmark Collection [12] and the National Library of Medicine [13]. We also evaluated the proposed method on data from another parasite, *Babesia*, to show the robustness of the DTGCN model. *Babesia* is a malaria-like parasite that infects RBCs and leads to the disease babesiosis [14]. With a ring-like structure, the ring forms of *Babesia* are sometimes confused with those of malaria parasites. The excellent results of the present study show that the proposed DTGCN is not only limited to the recognition of malaria parasites but can also effectively solve other microscopic image recognition problems. In general, the proposed DTGCN method can overcome the data variation and the imbalance problem in deep learning–based malaria recognition. Importantly, this DTGCN method can transfer a sufficiently trained recognition model to a completely unlabelled target dataset with unknown differences (such as colour, brightness, or imaging settings).

## Methods

### Data acquisition

The multi-stage malaria infected cell images were captured from blood smear samples stained with Giemsa reagent. This image set consists in total of 1,364 images at 1,000× magnification and is publicly available at the Broad Bioimage Benchmark Collection (BBBC) website [10]. All of the images were manually captured from *Plasmodium vivax*–infected patients in Manaus, Brazil, and Thailand under 1,000× magnification, annotated by 3 different experts globally. This dataset contains images from 2 classes of uninfected cells (RBCs and leukocytes) and 4 classes of parasitized cells (gametocytes, rings, trophozoites, and schizonts) with Giemsa stain. Although the initial purpose of this dataset was detection of parasitized cells rather than recognition of the various stages of malaria parasites, both bounding box coordinates and corresponding stage labels were provided. A total of 79,672 multi-stage parasitized and uninfected cell images were cropped from raw images according to the given box coordinates. As shown in Supplementary Table S1, numbers of each class are severely imbalanced—97.2% of them are 5,000 selected RBC images. Because the leukocyte class only contains 103 samples, we supplemented this class with 104 self-captured leukocyte images for testing. One hundred images were then randomly chosen from each class to form the testing dataset (600 samples in total) to evaluate the effectiveness of the proposed DTGCN, with the rest of the images used as training data. To avoid excessive consumption of computing resources by the imbalanced quantity of RBCs, 5,000 random RBCs were selected in experiments to save training time but achieve enough training efficiency. In total, this study used 7,456 microscopic images, consisting of 6,856 images for training and 600 images for testing. The details of the data distribution are provided in Supplementary Table S1. In each image, there is only 1 parasite or cell. Because the network accepts only image inputs of a certain pixel value, the input images with different numbers of pixels have been resized to 128 * 128 pixels before feeding into our deep learning model.

In addition, the second malaria parasite recognition task in this article is classification of unseen malaria parasites in a large-scale dataset that has different parameter distributions (such as brightness and imaging equipment settings) from the source training dataset. The BBBC dataset was adapted as the source domain in this multi-stage malaria parasite recognition task, and it was transferred to 2 binary classes for recognition: parasitized class (gametocytes, rings, trophozoites, and schizonts) and uninfected class (RBCs and leukocytes). This dataset, consisting of 13,780 testing images including both malaria parasites and RBCs [13], is available at the website of the National Library of Medicine . The images contain the segmented cells from Giemsa-stained thin blood smear slides of 150 *P. falciparum*–infected and 50 healthy patients under 1,000× magnification. Moreover, another 1,100 under-microscope *Babesia* and 1,100 RBC images were collected to validate the generalizability of our proposed DTGCN model

### Framework of DTGCN

**Figure 1:**
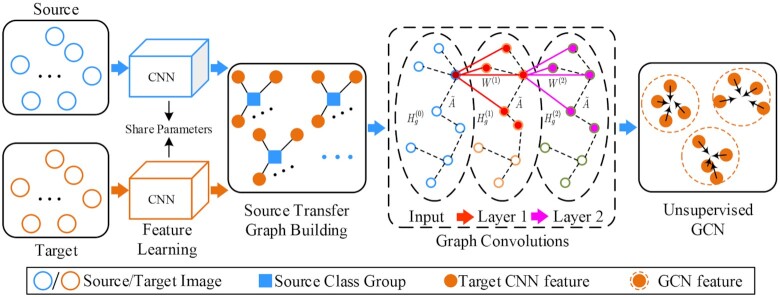
The schematic representation of DTGCN. First, the CNN-based feature extractor is in charge of learning representations from source and target data. Next, the source transfer graph building component connects target sample features to the source class groups by a proposed target-to-center source transfer graph building algorithm to formulate the graph topology correlations. Finally, the UGCN learns the graph representations by feeding CNN features and the formulated source transfer graph, which is jointly optimized by a contrastive classification loss in the source domain, and a maximum mean discrepancy (MMD) constraint for the feature-level transfer learning across 2 domains.

The DTGCN framework proposed for the recognition of multiple stages of malaria parasites consists of CNN feature learning, source transfer graph building, and the unsupervised graph convolutional network (UGCN). First, CNN is used to extract morphological features from images in each class. Second, a source transfer graph building algorithm is proposed to construct the class correlations between each source class group and target samples by a proposed target-to-center source transfer graph building algorithm according to the source class labels. Transferring the representatively discriminative information from the source into the target domain solves the challenges of dealing with parameter variations in an unfamiliar scenario. Then, the CNN features and source transfer graph are together fed into graph convolutional layers, which are optimized by ${L_{\mathrm{con}}}$ and ${L_{\mathrm{mmd}}}$ losses. Finally, we can conduct a *K*-means clustering algorithm on the final target graph feature representations and achieve the recognition of multi-stage malaria parasites in the target domain (Fig. 1).

### CNN feature learning

An obvious crucial preliminary step for image recognition is to extract representative morphological features. For the multi-stage malaria parasite recognition task, we use CNN as the backbone network owing to its superior performance in parasite microscopic image recognition [15], with excellent capability in dealing with challenging variations, such as illumination, background, and staining intensity. It can learn robust feature representations by multiplying overlapped convolutional operations with reasonable objective functions.

Mathematically, we assume the source data as ${X_s} = \{ x_1^s,x_2^s, \cdots ,x_{{N_s}}^s\} $ with complete labels ${Y_s} = \{ y_1^s,y_2^s, \cdots ,y_{{N_s}}^s\} $, and the target data as ${X_t} = \{ x_1^t,x_2^t, \cdots ,x_{{N_t}}^t\} $ without any labels, which contains the same categories as the source domain. To extract the appearance features by CNN, we define the backbone network as ${f_{\mathrm{cnn}}}$, to learn the CNN representations for source and target images. In this article, we use ResNet50 [16] architecture as the basic model of *f*_cnn_. Then, given the *i*th source image $x_i^s$ and *j*th target image $x_j^t$, the CNN features $h_i^s$ and $h_j^t$ can be calculated by

(1) \begin{equation*} h_i^s = {f_{\mathrm{cnn}}}(x_i^s;{\theta _{\mathrm{cnn}}}), \end{equation*}

 

(2) \begin{equation*} h_j^t = {f_{\mathrm{cnn}}}(x_j^t;{\theta _{\mathrm{cnn}}}), \end{equation*}

where $h_i^s \in {\mathbb{R}^d}$, $h_j^t \in {\mathbb{R}^d}$ (*d* is the dimension of feature vectors), and ${\theta _{\mathrm{cnn}}}$ is the learnable parameters in the backbone network*f*_cnn_. These features are the vectors of pre-softmax units, which attach a UGCN instead of prediction layers.

Importantly, to guarantee that the CNN features can obtain the identity information for source images, source feature vectors can be constrained by a contrastive loss function, denoted as CON loss,

(3) \begin{equation*} {L_{\mathrm{con}}} = \frac{1}{{2{N_s}}}\sum\nolimits_{i = 1}^{{N_s}} l {d^2} + (1 - l)\max {(m - d,0)^2}, \end{equation*}

where $d = {\| {h_i^s - h_k^s} \|_2}$ represents the Euclidean distance between 2 source features, $l = 1$ when $y_i^s = = y_k^s$, otherwise $l = 0$, and *m* is the margin setting among the distances. The contrastive loss is to maintain the representative information across different categories by learning a distance metric.

By this constraint, the backbone network ${f_{\mathrm{cnn}}}$ can learn discriminative feature representations only for source images. The remaining unmet challenge with major significance of this part is how to transfer the learnt knowledge into the target domain to bridge variations in the target scenario. To overcome this problem, a widely used strategy, maximum mean discrepancy (MMD), is introduced to constrain the learnt source and target features,

(4) \begin{equation*} {L_{\mathrm{mmd}}} = {\left\| {\frac{1}{{{N_s}}}\sum\nolimits_{i = 1}^{{N_s}} {h_i^s} - \frac{1}{{{N_t}}}\sum\nolimits_{j = 1}^{{N_t}} {h_j^t} } \right\|^2}. \end{equation*}

To further conduct the transfer learning and solve the problem of data imbalance, this article proposes a UGCN, in which the losses of contrastive classification and MMD are switched to create the constraint, rather than the aforementioned CNN features.

### Source transfer graph building

Generally, GCN provides an effective solution to simulate the correlations between objects in different distributions [17]. Thus, this article applies the transfer learning on GCN to alleviate the distribution gap between source and target domains, and to leverage the imbalanced data in the source domain to exploit the topological structure in the feature space. GCN can exploit the multi-stage malaria-infected cells by forwarding the message according to the node correlations–based adjacent matrix, which is one of the most important steps in GCN. This section aims to formulate the topological correlation graph as the base of the graph convolution layers.

Considering the imbalanced data in the source domain, the network transfers the class groups that contain the most representative information by class features. It will be recalled that there exist inherent correlations between the target images and the class groups in the source domain because they belong to the same classes. Inspired by this point, a source transfer graph building mechanism is designed by introducing the source class groups into the target domain.

By the CNN feature learning, the source and target image features (${H_s} = \{ h_1^s,h_2^s, \cdots ,h_{{N_s}}^s\} $, ${H_t} = \{ h_1^t,h_2^t, \cdots ,h_{{N_t}}^t\} $) are obtained by Eq. 1 and 2, respectively. For the features of the source images, the ${N_c}$ class centers $\{ h_c^n \in {\mathbb{R}^d}|_{n = 1}^{{N_c}}\} $ are calculated for each class by the following equation:

(5) \begin{equation*} h_c^n = \frac{1}{{\sum \nolimits_ {y_i^s = = n} }}\sum\nolimits_{i = 1}^{{N_s}} {(y_i^s = = n)h_s^i}. \end{equation*}

After computing the above, the network can obtain ${N_c}$ class centers of ${H_c} = \{ h_c^1,h_c^2, \cdots ,h_c^n, \cdots ,h_c^{{N_c}}\} $ for source image features, which are deployed into the graph construction. Meanwhile, this model implements *K*-means clustering on target feature vectors ${H_t}$. Assume an adjacent matrix $A \in {\mathbb{R}^{({N_s}{\mathrm{ + }}{N_t}) \times ({N_s}{\mathrm{ + }}{N_t})}}$ representing the correlations between the source and target samples. This framework uses the distance between target sample features and the source class center as the connected metrics. Specifically, given a target feature $h_j^t$, it is connected to the source class group when it has the smallest distance among all the source class groups,

(6) \begin{equation*} {{{\bf A}}_{kj}} = \left\{ {\begin{array}{@{}*{2}{l}@{}} {0,}&{{\mathrm{ if }}\arg \mathop {\min }\limits_n d(h_t^j,h_c^n) \ne y_k^s{\mathrm{ }}(1 \le n \le {N_c})}\\ {1,}&{{\mathrm{ if }}\arg \mathop {\min }\limits_n d(h_t^j,h_c^n) = = y_k^s{\mathrm{ }}(1 \le n \le {N_c})} \end{array}} \right. \end{equation*}

where $d( \cdot )$ is the Euclidean distance between target feature $h_t^j$ and source class center $h_c^n$. This formulation ensures that each target feature is connected with the nearest class group, and the framework can obtain the source transfer graph without any overlapped samples and connecting each other, as illustrated in Fig. 1.

Compared with other existing graph-building methods, the most significant difference is the first iteration of graph building. The traditional methods create the first graph on the basis of the CNN features, while the proposed method uses the features to iteratively formulate the new graph within each batch. The reason for this setting is that the learnt CNN feature provides discriminative information to the GCN, overcoming the imbalance problem in the image data.

### Unsupervised graph convolutional network

To strengthen the transfer learning ability of our model, we use a GCN to extract representation of each target feature and group source class groups in an unsupervised manner. It consists of a graph convolution stage and an unsupervised clustering objective function, which is in charge of malaria parasite recognition without any target annotation.

Given the source transfer graph in this article, the complete graph $G(V,A)$ can be formulated, where $V = \{ {v_1},{v_2}, \cdots ,{v_{{N_v}}}\} $ denotes the collection of the nodes with $|V| = ({N_s}{\mathrm{ + }}{N_t}) \times ({N_s}{\mathrm{ + }}{N_t})$, and $A \in {\mathbb{R}^{({N_s}{\mathrm{ + }}{N_t}) \times ({N_s}{\mathrm{ + }}{N_t})}}$ is the source transfer graph. Importantly, each node in this article contains a feature vector from the CNN backbone ${f_{cnn}}$, where the nodes can be replaced by an integrated feature set ${H_i}$ of ${H_i} = \{ h_1^s,h_2^s, \cdots ,h_{{N_s}}^s;h_1^t,h_2^t, \cdots ,h_{{N_t}}^t\} $, which is composed by ${N_s}$ source and ${N_t}$ target feature vectors. Thus, the graph can be redefined by $G({H_i},A)$. Because the GCN applied in a semi-supervised framework [17] has achieved a series of successes, the graph convolutional layers are optimized by the classification cross-entropy loss, which needs several labelled samples. However, the target data without any labels cannot work. Hence, the UGCN is proposed on the graph *G* without utilizing any target labels. The primary components in UGCN are the graph convolution layers in Fig. 1,

(7) \begin{equation*} H_g^{(l)} = {f_{\mathrm{gcn}}}(H_g^{(l - 1)},\tilde{A}) = \sigma ({\tilde{D}^{ - 1/2}}\tilde{A}{\tilde{D}^{ - 1/2}}{H^{(l - 1)}}{W^{(l)}}), \end{equation*}

where $l = 1,2, \cdots ,L$ denotes the *l*th graph convolution layer and *L* is the number of layers, and ${H^{(l - 1)}}$ and ${H^{(l)}}$ are the input and output graph features for the *l*th layer. In addition, $\tilde{A}$ represents the symmetrically normalized adjacent matrix with self-connections $(A + I)$, where *I* denotes the identity matrix. $\tilde{D}$ is the diagonal matrix of $\tilde{A}$, ${W^l}$ denotes the trainable weight parameters in the *l*th layer, and $\sigma $ is the non-linear activation, which is the ReLU function in this article. According to previously published work on GCN [18], the deeper GCN with multiple layers may be harmful to the graph feature learning. Therefore, this article also uses 2 graph convolutional layers to represent the final GCN features for each node, and the integrated CNN features are evolved as ${H_g} = \{ h_1^g,h_2^g, \cdots ,h_{{N_s}}^g;h_{{N_s} + 1}^g,h_{{N_s} + 2}^g, \cdots ,h_{{N_s} + {N_t}}^g\} $. The final representations for malaria parasite images are obtained by graph convolutions on the source transfer graph. To conduct the unsupervised recognition on the target GCN features, *K*-means clustering algorithm [19] is implemented to learn ${N_c}$ clusters $\{ {C_1}, \cdots ,{C_k}, \cdots ,{C_{{N_c}}}\} $ with ${N_c}$ feature collections $\{ {S_1}, \cdots ,{S_k}, \cdots ,{S_{{N_c}}}\} $, and to partition the learnt ${H_g}$, which is trained by ${L_{\mathrm{con}}}$, and ${L_{\mathrm{mmd}}}$ instead of the former CNN features; and the UGCN objective function for each cluster of *K*-means algorithm is as follows:

(8) \begin{equation*} {J_{\mathrm{ugcn}}}(k) = \frac{1}{{{N_t}}}\sum\nolimits_{i = {N_s} + 1}^{{N_s} + {N_t}} {\sum\nolimits_{h_i^g \in {S_k}} {{{\left\| {h_i^g - {C_k}} \right\|}^2}} }, \end{equation*}

where ${C_k}$ can be calculated by,

(9) \begin{equation*} {C_k} = \frac{1}{{|{S_k}|}}\sum\nolimits_{h_i^g \in {S_k}} {h_i^g}. \end{equation*}

It should be emphasized that the *K*-means clustering algorithm has excellent capability in calculating cluster centers in Euclidean distance space, and it can aggregate similar features by their Euclidean metrics. Here, we also train the whole network (CNN and GCN) by ${L_{\mathrm{con}}}$ and ${L_{\mathrm{mmd}}}$ jointly after the feature extraction of GCN, rather than working on the former CNN features. In detail, the updating of *K*-means is conducted on the learnt GCN features (Supplementary Algorithm S1). Thus, this network can achieve a satisfactory unsupervised classification on the learnt graph features after being fully trained.

### Network training and evaluation metrics

We present the details of network training, evaluation metrics, and compared models in Supplementary Fig. S4.

To demonstrate the superior effectiveness of our DTGCN models, we chose 3 widely used deep learning networks, Visual Geometry Group Network (VggNet) [20], the GoogLe Inception V3 Network (GoogLeNet) [21], and the deep Residual Network (ResNet) [16], to be the contrasts. In addition, we adopted 4 recently proposed malaria parasite recognition methods [22,23,24,25] with fine-tuning in our experimental datasets to conduct efficient comparison, and the proposed DTGCN was also modified to evaluate the core components of feature learning, graph-building algorithm, and the UGCN. The modifications were used to build 2 updated models: (i) removing the GCN by directly attaching graph features to the *K*-means algorithm on the target CNN features (denoted as Baseline), and (ii) using a common *K*-nearest neighbours (KNN) algorithm to formulate the graph (Ours+KNN). On the other hand, this study also evaluates the influence of the deeper CNN in feature learning, which is explored by changing the depth of ResNet: ResNet18, ResNet34, and ResNet50 were tested individually. Thus, we evaluated 2 more models (Ours+ResNet18 and Ours+ResNet34).

## Results

### Performance on multi-stage malaria parasite recognition

To validate the effectiveness of the DTGCN, this study first implemented extensive experiments on multi-stage malaria parasite recognition. We report the training and evaluation of several different models (VggNet, GoogLeNet, ResNet, Quinn et al. [22], Rajaraman et al. [23], Vijayalakshmi [24], Umer et al. [25], and DTGCN) in this section, and their performance is presented in the first 12 lines of Table 1. As for the baselines of recent papers, Quinn et al. [22] designed a deep learning model trained from annotated cell images with 4 hidden layers consisting of 2 convolution layers, 1 pooling layer, and a fully connected layer. The performance of the deep neural networks was evaluated on the detection of malaria parasites in thick blood smear samples, revealing an average precision of 97%. Rajaraman et al. [23] used pre-trained CNN-based deep learning models as feature extractors to classify parasitized and uninfected cells, obtaining 95.7% classification accuracy on single-stage malaria detection. Vijayalakshmi [24] developed a novel transfer learning approach to identify cells infected with malaria parasite, which is powered by combining VggNet and support vector machine. The results on malaria digital corpus images achieved a classification accuracy of 93.1%. Umer et al. [25] applied pre-processing steps for re-sampling and normalizing input microscopy images and then utilized stacked CNN by fine-tuning it along with max-pooling and dropout layer. The performance of this model was evaluated on single stage malaria parasite detection, resulting in 99.98% accuracy. Specifically, these baseline methods were re-trained on the BBBC dataset and tested on the multi-stage malaria parasite images. The results show that the proposed DTGCN achieves an excellent performance, with overall accuracy of 98.3%; moverover, the precision, recall, and F1-score are all >98%. Comparing with other CNN models that have been used, the proposed DTGCN had clearly superior performance because the best among the other 3 models (GoogLeNet) only realized ∼83% accuracy in the same task. Compared to DTGCN, recent publications have tested their methods only for single-stage malaria parasite recognition, leaving these methods vulnerable to the variations in multi-stage malaria parasite recognition. The variations degrade the performance of the previously developed models, restricting them to a maximum recognition accuracy of 66.3%. Therefore, a technique consisting of simple adaptation of existing single-stage classification models into multi-stage malaria parasite recognition will perform poorly. In a practical scenario, non-RBC images are often captured at a small scale, which is a result of the complicated image-capturing operations involved. This is one of the most challenging problems in deep learning applications because deep learning requires a large amount of data. In this article, our model can train a robust feature learning network with only small number of non-RBC images, while existing deep learning methods cannot handle this challenging problem. This reveals the excellent capability of our model on the multi-stage malaria parasite recognition task.

**Table 1:** Performance on multi-stage malaria parasite recognition

Method	Accuracy	Precision	Recall	F1-score
**Performance overall (%)**				
VggNet	77.3 ± 0.53	81.0 ± 0.44	77.3 ± 0.28	76.8 ± 0.23
GoogLeNet	82.7 ± 0.31	85.9 ± 0.29	82.7 ± 0.25	83.0 ± 0.18
ResNet	81.0 ± 0.28	87.7 ± 0.21	81.0 ± 0.19	81.5 ± 0.20
Quinn et al. [22]	60.3 ± 0.48	77.1 ± 0.36	60.3 ± 0.30	55.1 ± 0.34
Rajaraman et al. [23]	61.5 ± 0.35	78.0 ± 0.28	61.5 ± 0.31	56.9 ± 0.20
Vijayalakshmi [24]	66.3 ± 0.29	79.0 ± 0.26	66.3 ± 0.23	63.0 ± 0.23
Umer et al. [25]	30.0 ± 0.32	43.1 ± 0.37	30.0 ± 0.42	16.4 ± 0.30
Baseline	80.9 ± 0.42	86.3 ± 0.42	81.4 ± 0.10	81.9 ± 0.18
Ours+KNN	78.7 ± 3.58	72.9 ± 3.21	78.7 ± 2.94	74.4 ± 3.21
Ours+Res18	95.0 ± 0.08	95.1 ± 0.11	95.0 ± 0.14	95.0 ± 0.09
Ours+Res34	96.7 ± 0.09	97.2 ± 0.10	96.7 ± 0.04	96.6 ± 0.05
**Ours+Res50**	**98.3 ± 0.03**	**98.5 ± 0.02**	**98.3 ± 0.02**	**98.3 ± 0.03**
**Performance of Ours+Res50 on each stage (%)**				
Gametocyte	99.6 ± 0.04	91.7 ± 0.05	99.8 ± 0.02	95.6 ± 0.04
Leukocyte	99.8 ± 0.02	99.9 ± 0.01	99.7 ± 0.01	99.8 ± 0.02
RBC	99.9 ± 0.01	99.9 ± 0.01	99.9 ± 0.02	99.9 ± 0.04
Ring	99.9 ± 0.01	99.8 ± 0.02	99.7 ± 0.03	99.7 ± 0.02
Schizont	90.8 ± 0.05	99.9 ± 0.03	90.8 ± 0.05	95.1 ± 0.06
Trophozoite	99.8 ± 0.02	99.8 ± 0.02	99.9 ± 0.01	99.8 ± 0.02

Note: Proposed DTGCN method with the best results is boldfaced.

In addition, DTGCN classification of each stage of the intra-erythrocytic cycle of malaria is reported in the lower part of Table 1. For single-stage classification, this model has the best performance in classifying most stages, and all the indicators are >99%. Meanwhile, the classification of the schizont stage is slightly worse than the others but still ∼90.8%; the schizont stage is more likely to be misclassified. This might be because the schizont is similar to the gametocyte, as illustrated in Fig. S1.

**Figure 2:**
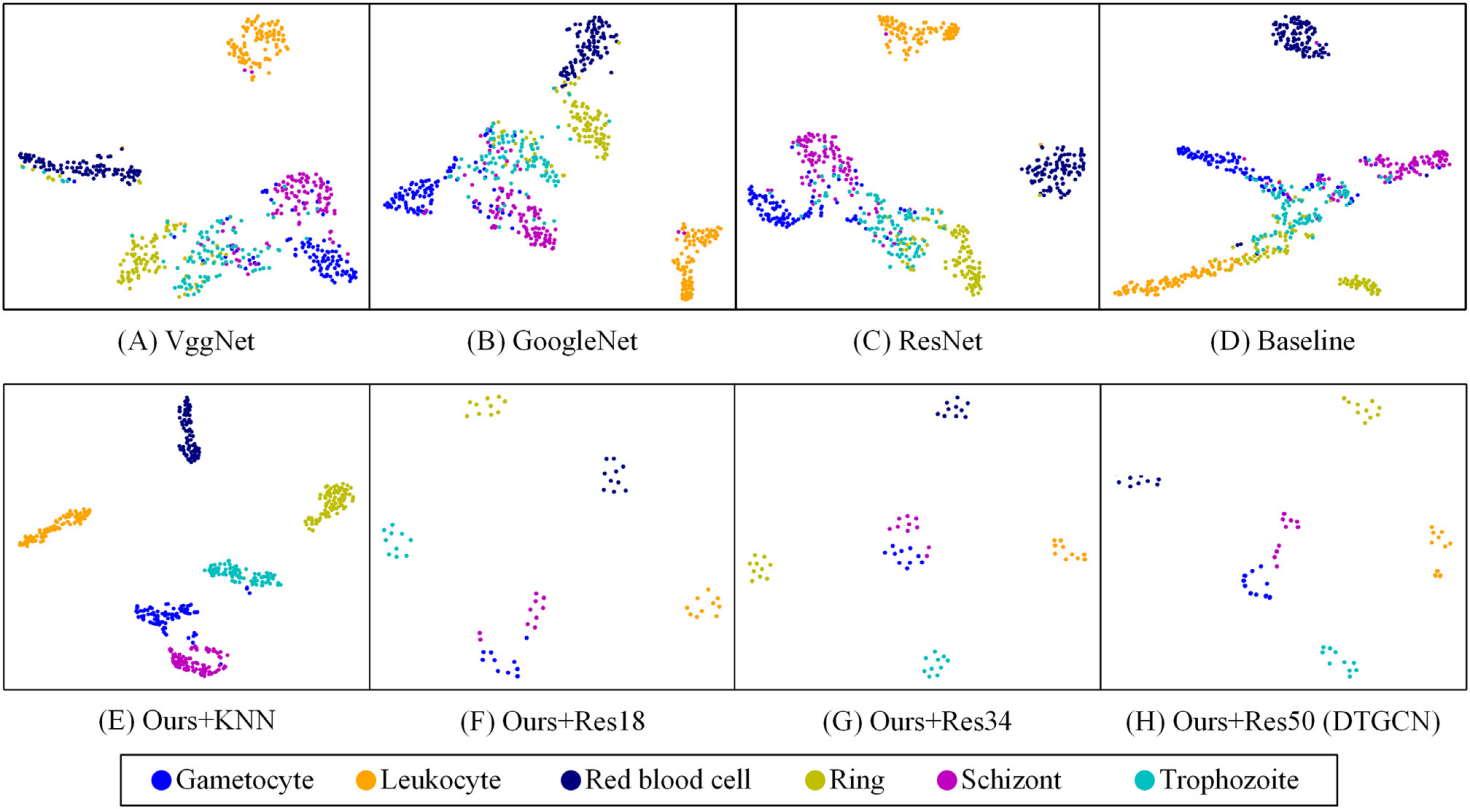
t-SNE performance on malaria parasite recognition. The t-SNE plots of VggNet (a), GoogLeNet (b), ResNet (c), and Baseline (d) are compared to various DTGCN approaches, including replacing the graph building graph by KNN algorithm (Ours+KNN) (e) and replacing the CNN backbones of ResNet18 (Ours+Res18) (f), ResNet34 (Our+Res34) (g), and ResNet50 (Ours+Res50) (h). t-SNE plots provide a method to evaluate and refine clustering of each class of sample images. Data points are coloured according to their categories, and many points are overlapped in (f–h). DTGCN achieves the best t-SNE plot performance compared to other baselines. The t-SNE plot shows that Ours+Res50 is the best discriminated.

Additionally, the 2D t-distributed stochastic neighbour embedding (t-SNE) plot is deployed to show the clustering performance in Fig. 2h to visualize the capacity of the models in distinguishing multi-stage malaria parasites and uninfected cells. The t-SNE can be used to visualize high-dimensional data in 2 dimensions, maintaining local structures. In t-SNE, pairs of points are given joint probabilities based on their distance, and the Kullback-Leibler divergence between the probabilities is minimized [10]. The t-SNE plots of 8 models are shown in Fig. 2. Generally, the difference between multi-stage malaria parasites in infected and uninfected cells is clear and easy to distinguish. Specifically, Fig. 2h shows that the proposed DTGCN can learn the features that have less intra-class distance and larger cross-class distance, which means that the same-class samples are clustered and the margins between multiple stages and uninfected samples are far enough to be distinguished easily by the following classifying procedure. However, the other-models-learnt features have insufficient cross-class distances. For example, the Vgg model (Fig. 2a) has especially unclear clusters and this model has the worst performance in classification.

**Figure 3:**
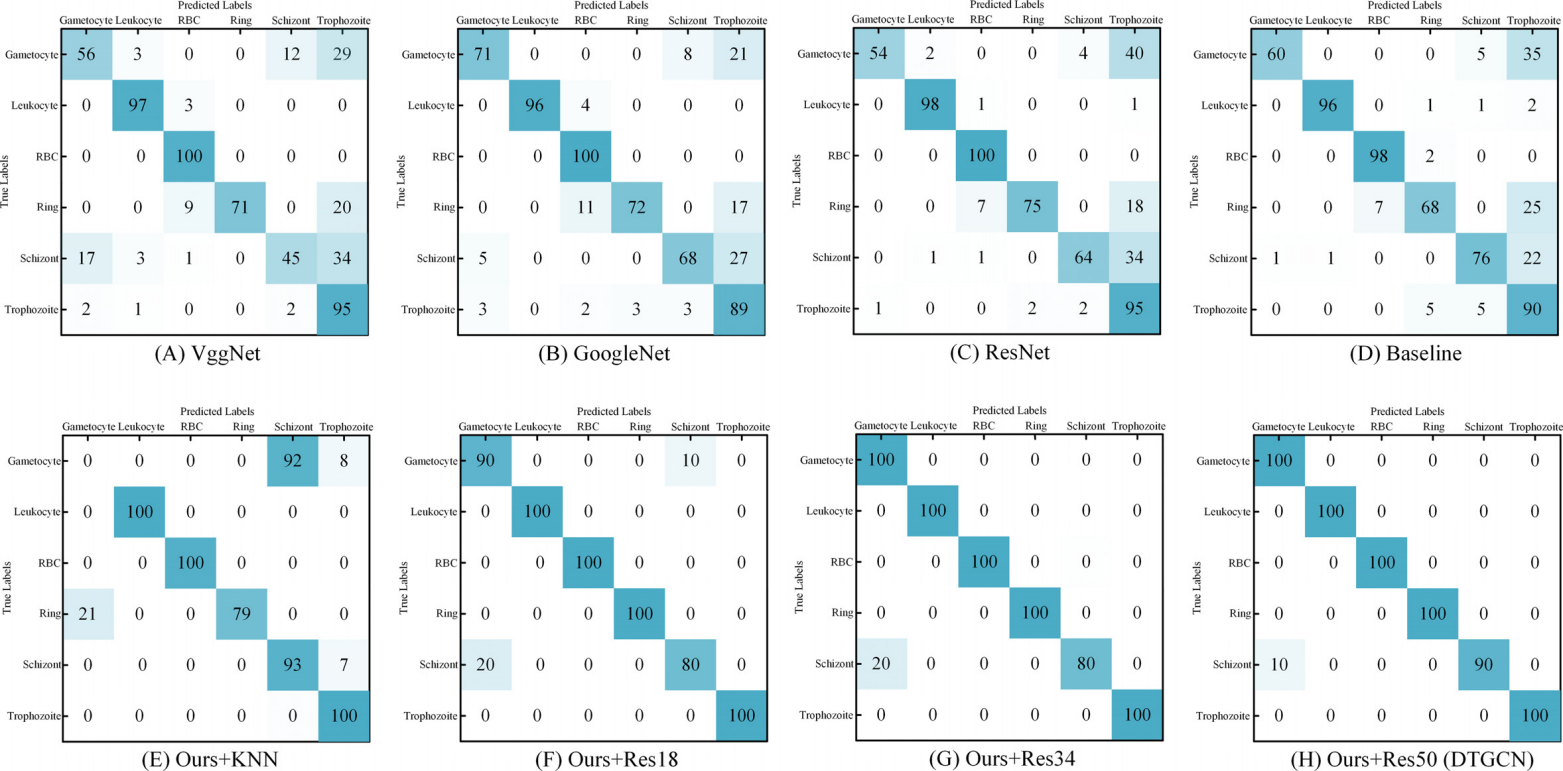
Confusion matrixes for the multi-stage malaria parasite classification. The confusion matrices of VggNet (a), GoogLeNet (b), ResNet (c), and Baseline (d) are compared to various DTGCN approaches, including replacing the graph-building graph by KNN algorithm (Ours+KNN) (e) and replacing the CNN backbones by ResNet18 (Ours+Res18) (f), ResNet34 (Our+Res34) (g), and ResNet50 (Ours+Res50) (h). Confusion matrix reveals the variation in misclassification between the predicted and true labels. The diagonal cells correspond to samples that are correctly classified. The off-diagonal cells correspond to incorrectly classified samples. It is easy to see that Ours+Res50 (DTGCN) presents the best classification results on the confusion matrix.

To better reveal the recognition result on the 600 testing images, this study uses the confusion matrix to visualize the accuracy of the multi-stage malaria parasite recognition in Fig. 3h. The confusion matrix reveals the variation in misclassification between each class, and each column of the matrix represents the predicted class, where the summation of the column is equal to the predicted number of images in this class. Each row in this matrix denotes the true classes, and their summation is the total number of real images in this class. From Fig. 3h, it can be observed that the proposed DTGCN only misclassifies 10 out of 600 total images tested. To sum up, DTGCN identifies malaria parasites effectively in multiple stages, and the DTGCN would be especially useful for humans.

### Performance on a large-scale dataset of malaria parasite recognition

To further validate the DTGCN's performance on a larger malaria parasite dataset, the trained models of first 7 comparative methods from the previous subsection were directly tested on a larger-scale dataset that consists of 13,780 testing images with an equal amount of infected and uninfected images. The task was binary classification. To keep the classes consistent, the gametocyte, ring, schizont, and trophozoite stages were grouped as the parasitized cells, and leukocytes and RBCs were considered uninfected cells to conduct the experiment. All models trained on the multi-stage malaria parasite images were directly transferred and tested on the large-scale malaria recognition dataset without further training. As reported in Table 2, our DTGCN achieved ∼95% in accuracy, F1-score, precision, and recall, compared to VggNet (72.4%), GoogLeNet (74.4%), ResNet (72.3%), Quinn et al. [22] (51.1%), Rajaraman et al. [23] (67.1%), Vijayalakshmi [24] (66.0%), Umer et al. [25] (61.9%). These methods keep consistency with our DTGCN on Jaeser dataset following transfer learning strategy, i.e., we train them only on source dataset and directly apply the models on target datasets. And VggNet still has the relative worse performance, which is consistent with the previous experiment. In addition, there is a clear separation between infected and uninfected cells in the t-SNE plot Fig. S2 h. Contrarily, the VggNet has the most mixed t-SNE with the worst classification results. This illustrates that the separable features benefit the classification and this is exactly the operation that the proposed DTGCN completes. Importantly, DTGCN does not require any target image labels. This property efficiently solves the problem of the lack of large-scale labelled data, which is caused by the high time consumption of sufficient under-microscope labelling, which is a labor-intensive process that can only be performed by well-trained professionals. The corresponding confusion matrixes are also displayed in Fig. S3.

**Table 2:** Performance on the large-scale malaria parasite dataset

Method	Accuracy	Precision	Recall	F1-score
**Performance overall (%)**				
VggNet	72.4 ± 0.23	74.2 ± 0.19	72.4 ± 0.22	71.8 ± 0.27
GoogLeNet	74.4 ± 0.22	81.0 ± 0.18	74.4 ± 0.23	72.9 ± 0.21
ResNet	72.3 ± 0.16	81.6 ± 0.18	72.2 ± 0.18	70.1 ± 0.17
Quinn et al. [22]	51.1 ± 0.28	55.0 ± 0.25	51.19 ± 0.29	39.2 ± 0.23
Rajaraman et al. [23]	67.1 ± 0.31	67.6 ± 0.29	37.1 ± 0.33	66.8 ± 0.30
Vijayalakshmi [24]	66.0 ± 0.27	33.9 ± 0.21	66.0 ± 0.24	65.3 ± 0.25
Umer et al. [25]	61.9 ± 0.31	64.8 ± 0.28	61.9 ± 0.29	43.8 ± 0.22
Baseline	74.9 ± 0.17	74.9 ± 0.25	74.9 ± 0.17	74.9 ± 0.19
Ours+KNN	86.4 ± 2.35	87.3 ± 3.10	86.4 ± 2.84	86.3 ± 2.64
Ours+Res18	91.9 ± 0.10	92.1 ± 0.09	91.9 ± 0.08	91.9 ± 0.12
Ours+Res34	93.6 ± 0.08	93.6 ± 0.07	93.6 ± 0.09	93.6 ± 0.08
**Ours+Res50**	**95.4 ± 0.05**	**95.4 ± 0.07**	**95.4 ± 0.06**	**95.4 ± 0.07**
**Performance of Ours+Res50 on each class (%)**				
Parasitized	96.1 ± 0.06	94.8 ± 0.07	96.1 ± 0.05	95.4 ± 0.06
Uninfected	94.7 ± 0.04	96.0 ± 0.07	94.7 ± 0.07	95.4 ± 0.07

Note: Proposed DTGCN method with the best results is boldfaced.

This binary classification task based on large-scale data demonstrates that the DTGCN method not only has superior performance in recognizing multi-stage malaria parasites but also works well in an unseen large-scale malaria dataset. This extraordinary capacity saves the expensive labelling work in biomedical image analysis and provides a novel method for unlabelled biomedical image classification. This part shows that DTGCN outperforms other alternative methods on the unseen large dataset of malaria parasites and it further demonstrates the generalizability of DTGCN in malaria parasite recognition.

### Performance on babesia parasite recognition

As mentioned in the Introduction, *Babesia* infects RBCs and leads to the disease babesiosis. Because the clinical and laboratory presentations of babesiosis and malaria are similar (ring-like structure), the ring forms of *Babesia* are sometimes confused with those of the malaria parasite. Thus, a *Babesia* parasite recognition test was conducted in this study to distinguish between *Babesia* parasites and RBCs to evaluate the discriminant ability of the proposed model for other parasites. With the similar ring shapes within infected RBCs, the recognition task was also challenging for transfer learning. Surprisingly, the proposed DTGCN achieved 99.0% accuracy, 99.01% precision, 99.0% recall, and 98.99% F1-score when training the model on 600 *Babesia* and 600 RBC images. The reason that this model can distinguish *Babesia* parasites and RBC might be the fact that DTGCN can successfully overcome the problem of insufficient and imbalanced training data. This experiment shows that the proposed DTGCN is effective, flexible, and scalable when presented with a challenging microscopic object recognition task.

### Further analysis of our DTGCN

The proposed DTGCN for multi-stage malaria recognition is composed of 3 major modules: CNN feature learning, target-to-center source transfer graph building, and the UGCN. In this section, we discuss the capacities of each main component of this model and explore the reasons why this collaborative model works so well.

#### Analysis of CNN feature learning module

To analyse the contribution of CNN feature learning and evaluate the influence of CNN depth, we extracted CNN features by different ResNets with 18, 34, and 50 layers. The relative recognition results are summarized in Tables 1 and 2 for multi-stage malaria parasites and large-scale binary malaria parasite images, respectively; t-SNE and confusion matrices are also illustrated in Figs 2 and 3 and Supplementary Figs S2 and S3, respectively. Here, DTGCN achieved the best accuracy when the model used ResNet50, rather than the shallow networks of ResNet18 and ResNet34, as the CNN feature extractor because the deeper network usually has better feature learning ability for image classification. Taking multi-stage malaria parasite recognition as an example, DTGCN (Ours+Res50) obtained 98.3% accuracy, while Ours+Res18/34 achieved 95.0%/96.7%. It is easy to see that the deeper CNN generates the higher accuracy and shallower CNNs achieve weaker performance (ResNet50 > ResNet34 > ResNet18).

#### Analysis of source transfer graph building module

As the major step of GCN, the graph-building algorithm is extremely important to establish a natural and topological structure for GCN. This article involves a novel graph-building mechanism, named "target-to-center source transfer graph building algorithm," which connects the target image features to the source class groups by measuring the distance between each sample's cluster and source class centers.

To show the contribution of the proposed graph-building algorithm for multi-stage malaria parasite recognition, we deploy a well-known KNN graph-building algorithm, which is usually used in GCN, to replace our module. This modified method, named "Ours+KNN" in Tables 1 and 2, produced accuracies of only 78.7% and 86.4% for multi-stage and binary malaria parasite recognition. The absolute superiority of the performance on t-SNE and confusion matrixes in Figs 2 and 3 and Supplementary Figs S2 and S3 of the proposed source transfer graph-building algorithm proves that the established GCN is a reasonable and robust topological method for solving the transfer learning problem in microscopic image recognition.

#### Analysis of unsupervised graph convolutional network

The crucial module of DTGCN is the proposed unsupervised GCN, which aims to explore the correlations between the unlabelled target samples given the inputs of CNN features and graph. To comprehensively evaluate the contribution of UGCN, we removed this module from DTGCN and directly utilized the *K*-means algorithm on the target CNN features, which were extracted from the sufficiently trained CNN feature learning module. This modification is defined as the Baseline of the proposed DTGCN, and the detailed results on BBBC datasets are reported in Table 1. From the comparison of DTGCN and Baseline, the UGCN improves the accuracy by 17.4% (from 80.9% to 98.3%), as well as greatly increasing in other metrics. From that, the proposed DTGCN appears to result in an overall preponderance over Baseline, because of the contribution of UGCN. To further demonstrate the benefit from UGCN, this article also visualizes the t-SNE of Baseline on multi-stage malaria parasite recognition (Fig. 2d) and large-scale malaria parasite data (Supplementary Fig. S2d), as well as confusion matrices (Fig. 3d and Supplementary Fig. S3d). In terms of the recognition results and the visualization comparison, the significant contribution of UGCN on exploiting the discriminant topological correlations is fully presented and proved.

#### Influence of the training data size (in source domain)

In general transfer learning tasks, the source data play an important role in the domain adaptation, and the efficacy of the transferred model is most reliant on the scale of the source data. To evaluate the influence of training data size in our DTGCN, different data sizes from the source domain were randomly selected from each class with equal percentages (20%, 40%, 60%, 80%, 100%) to conduct transfer learning on the target multi-stage malaria parasite dataset. In Supplementary Fig. S4, the box plot visualizes the accuracy results by conducting 5 randomly repeated iterations. The plot shows that DTGCN realizes 89.3% and 97.7% accuracy when utilizing 20% and 40% source data, respectively. Furthermore, DTGCN achieves an acceptable accuracy of 97.7% when using 60% of source data, instead of using the entire source dataset. This demonstrates that the proposed DTGCN is trainable with limited data. This analysis concludes that the more source training data, the better the transfer learning performance that will be achieved. And this result suggests that researchers should use as much source data as possible to support the transfer learning on the target domain.

## Discussion and Conclusion

To our knowledge, this study is the first to investigate multi-stage malaria parasite recognition with the use of a deep transfer graph convolutional network (DTGCN) approach. In this article, the DTGCN consists of 3 major components: CNN for feature learning, source transfer graph building, and unsupervised GCN.

We designed the source transfer graph building and UGCN for multi-stage parasite recognition, aiming to solve the problems of data variations and imbalanced data. With this knowledge, the transfer learning between labelled source data and unlabelled target data can work in many scenarios. The proposed model first learns the CNN features by a ResNet architecture with constraints of MMD and contrastive losses. It then utilizes the target-to-center–based source transfer graph-building algorithm to connect the source class groups with target samples, to leverage the imbalanced data. After CNN feature learning and graph building, DTGCN uses UGCN to further alleviate the feature distribution gap between source and target domains with a *K*-means algorithm. Thus, the proposed framework can achieve multi-stage malaria parasite recognition results by the *K*-means algorithm on the final graph feature representations from the target domain. What needs to be emphasized is that the proposed DTGCN not only works out the supervised multi-stage malaria parasite recognition task but also does not require any microscopic image labels in the target domain, which means that this model can be transferred to solve an unseen scenario to conduct recognition tasks. The proposed DTGCN can also be applied to other biomedical image recognition tasks that have complicated procedures for data collection and annotation.

Through experiments on publicly available multi-stage and binary microscopic malaria parasite images, this article has successfully demonstrated that a DTGCN model can extract information to boost the accuracy of deep learning. Results on malaria-like *Babesia* parasites show that a DTGCN model can also be used for detecting other parasites under microscope.

The proposed method for multi-stage malaria parasite microscopic image analysis can be immensely helpful in the development of a low-cost, automated malaria diagnostic solution. This can significantly improve efficiency and reduce the need for dedicated pathologists in areas with limited resources.

## Data Availability

The *P. vivax* (malaria) infected human blood smear data are available in the BBBC repository and can be accessed with accession No. BBBC041. The large scale malaria dataset consisting of 13,780 both malaria parasites and RBCs testing images are available in the National Library of Medicine (NLM) respository and can be accessed with accession No. PUB9932. Snapshots of our code and other data further supporting this work are openly available in the *GigaScience* repository, GigaDB [28].

## Availability of Supporting Source Code and Requirements

Project name: Deep Transfer Graph Convolutional Network

Project home page: https://github.com/senli2018/DTGCN_2021

Operating system(s): Windows 10 with Nvidia Geforce 2080Ti GPU

Programming language: Python 3.6.0

Other requirements: Pytorch 1.0.0 or higher, Torchvision 0.4.1 or higher, Scipy 1.1.0 or higher, and Numpy 1.17.4 or higher

License: MIT

RRID:SCR_020976

Biotools: deep_transfer_graph_convolutional_network

BBBC041: https://data.broadinstitute.org/bbbc/BBBC041

PUB9932: https://lhncbc.nlm.nih.gov/publication/pub9932

## Additional Files

**Supplementary Figure S1**. The life cycle of malaria parasites. (a) The intra-erythrocytic cycle of malaria parasites. (b) Examples of multi-stage malaria parasites. Malaria parasites undergo several stages in their complex life cycle. The malaria parasites undergo repeated rounds of asexual multiplication (the intra-erythrocytic developmental cycle). During the intra-erythrocytic cycle, parasites go through the ring, trophozoite, and schizont stages. In each cycle, a small proportion of parasites begin to develop into the sexual form of the parasite, which is known as a gametocyte.

**SupplementaryFigure S2**. t-SNE performance on large-scale malaria parasite binary classification. The t-SNE plots of VggNet (a), GoogleNet (b), ResNet (c), and Baseline (d) are compared to various DTGCN approaches, including replacing the graph-building graph by KNN algorithm (Ours+KNN) (e) and replacing the CNN backbones with ResNet18 (Ours+Res18) (f), ResNet34 (Our+Res34) (g), and original ResNet50 (Ours+Res50) (h). The t-SNE plots provide a method to evaluate and refine the clustering of each class of sample images. Data points are coloured according to their categories. The performance on large-scale malaria parasite classification is similar to the multi-stage parasite classification, showing that Ours+Res50 is the best discriminated.

**Supplementary Figure S3**. Confusion matrices for the multi-stage malaria parasite classification. The confusion matrices of VggNet (a), GoogLeNet (b), ResNet (c), and Baseline (d) are compared to various DTGCN approaches, including replacing the graph building graph by KNN algorithm (Ours+KNN) (e) and replacing the CNN backbones with ResNet18 (Ours+Res18) (f), ResNet34 (Our+Res34) (g), and ResNet50 (Ours+Res50) (h). The confusion matrix reveals the variation in misclassification between the predicted and true labels. The diagonal cells correspond to samples that are correctly classified. The off-diagonal cells correspond to incorrectly classified samples. It is easy to see that Ours+Res50 (DTGCN) presents the best classification results on the confusion matrix.

**Supplementary Figure S4**. The impact of source data size for recognition accuracy. The DTGCN model is trained by increasing the numbers of source examples as reported in percent of original size (6,856). Note that the percentage of used images preserves equivalent ratios of RBC to non-RBC images. For every reported training data size, 5 repeat trainings are performed. The accuracies are calculated and reported as box plots (n = 5). The results of this study support the fact that a large number of training images (≥40%) are necessary for good performance (accuracy >90%). This visualization reveals that dataset size plays an important role in achieving high accuracy in classification.

**Supplementary Figure S5**. Visualization of several convolutional feature maps learned by the top 3 layers of DTGCN. To show the evidence of feature detection in more challenging use case examples, we randomly visualize their feature maps from the top 3 convolutional layers. A feature map generated from convolutional layers can reveal the detailed feature-learning procedure in the deep learning method. The feature map visualization demonstrates that our DTGCN can extract clear morphological features from these selected challenging images, which can prove that our DTGCN has excellent capability in feature representation for challenging multi-stage malaria parasite recognition.

**Supplementary Table S1**. The details of the multi-stage malaria parasite images used in this study. The training images of multi-stage parasites had imbalanced class distribution, where most images captured under the microscope were red blood cells. This table illustrates the numbers of training and test data.

**Supplementary Table S2**. Experimental settings of the compared methods on multi-stage malaria parasite recognition. Details regarding maximum epoch number, batch size for source/target data, learning rate, and optimizer are summarized. The learning rates of each method are initialized with the value in Table S2 and will multiply by 0.1 in each of 50 epochs along with the training. In the modified methods of DTGCN, the maximum epoch number is 50, learning rate is 1e−5, and optimizer is Adam for our network.

**Supplementary Table S3**. Experimental settings of the compared methods on large-scale malaria parasite recognition. Details regarding maximum epoch number, batch size for source/target data, learning rate, and optimizer are summarized. The learning rates of each method are initialized with the value in Table S3 and will multiply by 0.1 in each of 50 epochs along with the training. In the modified methods of DTGCN, the maximum epoch number is 50, learning rate is 1e−5, and optimizer is Adam for our network.

**Supplementary Algorithm S1**. Deep Transfer Graph Convolutional Network Algorithm. This algorithm uses a DTGCN to alleviate the domain gap between the source and target domains to solve the class-imblance problem. This algorithm employs $X_s$, $Y_s$ as input source data and $X_t$ as target data. Then the network is optimized using losses of CON and MMD with the initialization of parameters in *M* iterations. Finally, the target images $X_t$ is tested by conducting *K*-means algorithm on the learned GCN features.

## Abbreviations

CNN: convolutional neural network; GCN: graph convolutional network; GPU: graphics processing unit; KNN: *K*-nearest neighbours; MMD: maximum mean discrepancy; RBC: red blood cell; t-SNE: t-distributed stochastic neighbour embedding.

## Funding

The project is financially supported by the Natural Science Foundation of Shenzhen City (Project No. JCYJ20180306172131515) and the Fundamental Research Funds for the Central Universities (Project No. HIT.NSRIF.2020064).

## Competing Interests

The authors declare that they have no competing interests.

## Authors' Contributions

Y.Z. contributed to the supervision of the study. Y.Z. and S.L. contributed to the design of the study. S.L., Z.D., and X.M. performed data collection and analysis. All authors contributed to literature review, writing, and critical revision of the manuscript.

## Supplementary Material

giab040_GIGA-D-21-00019_Original_Submission

giab040_GIGA-D-21-00019_Revision_1

giab040_GIGA-D-21-00019_Revision_2

giab040_GIGA-D-21-00019_Revision_3

giab040_Response_to_Reviewer_Comments_Original_Submission

giab040_Response_to_Reviewer_Comments_Revision_1

giab040_Response_to_Reviewer_Comments_Revision_2

giab040_Reviewer_1_Report_Original_Submission Barath Narayanan -- 1/31/2021 Reviewed

giab040_Reviewer_1_Report_Revision_1 Barath Narayanan -- 3/7/2021 Reviewed

giab040_Reviewer_2_Report_Original_Submission Michael Rung-Tsong Lyu -- 2/12/2021 Reviewed

giab040_Reviewer_3_Report_Original_Submission Chris Armit -- 2/15/2021 Reviewed

giab040_Reviewer_3_Report_Revision_1 Chris Armit -- 3/4/2021 Reviewed

giab040_Reviewer_3_Report_Revision_2 Chris Armit -- 3/23/2021 Reviewed

giab040_Supplemental_File
