# Supplementary material for: Multi-stage malaria parasite recognition by deep learning
Source: Gigascience. 2021 Jun 17;10(6):giab040. doi: 10.1093/gigascience/giab040 (PMC8210472; doi:10.1093/gigascience/giab040)
Supplement: giab040_GIGA-D-21-00019_Revision_2 [file giab040_giga-d-21-00019_revision_2.pdf]

GigaScience

Multi-Stage Malaria Parasites Recognition by Deep Learning

--Manuscript Draft--

**Manuscript Number:**	GIGA-D-21-00019R2
**Full Title:**	Multi-Stage Malaria Parasites Recognition by Deep Learning
**Article Type:**	Research
**Funding Information:**	Science and Technology Planning Project of Shenzhen Municipality (JCYJ20180306172131515) — Dr Yang Zhang
**Abstract:**	Motivation: Malaria, a mosquito-borne infectious disease affecting humans and other animals, is widespread in the tropical and subtropical regions. Microscopy is the most common method in diagnosing the malaria parasite from stained blood smears. However, the widespread manually under-microscope parasite examining is time-consuming, error-prone and well-trained-professional required. Besides, the recognition of a malaria parasite through microscope is still a challenging process, especially in distinguishing multiple stages of parasites.<br>Results: In this paper, we develop a novel deep learning approach for multi-stage malaria parasites recognition in blood smear images using Deep Transfer Graph Convolutional Network (DTGCN). And this paper is the first application of GCN on multi-stage malaria parasites recognition in blood smear images. The proposed DTGCN model is based on unsupervised learning by transferring knowledge learnt from source images that contain the discriminative morphology characteristics of multi-stage malaria parasites. These transferred information guarantees the effectiveness on the target parasites recognition. This approach firstly learns the identical representations from source to establish the topological correlations between source class centers and the unlabelled target samples. In this stage, the Graph Convolutional Network (GCN) is implemented to extract graph feature representations for the multi-stage malaria parasites recognition. The proposed method showed the high-er accuracy and effectiveness in publicly available microscopic images of multi-stage malaria parasites compared to a wide range of state-of-the-art approaches. Furthermore, this method is also evaluated on a large-scale dataset of unseen malaria parasites and the Babesia dataset.<br>Availability: Code and dataset available at https://github.com/senli2018/DTGCN under an OSI compliant license (MIT).
**Corresponding Author:**	Yang Zhang<br>Harbin Institute of Technology Shenzhen<br>Shenzhen, China CHINA
**Corresponding Author Secondary Information:**	
**Corresponding Author's Institution:**	Harbin Institute of Technology Shenzhen
**Corresponding Author's Secondary Institution:**	
**First Author:**	Sen Li
**First Author Secondary Information:**	
**Order of Authors:**	Sen Li<br>Zeyu Du<br>Xiangjie Meng<br>Yang Zhang
**Order of Authors Secondary Information:**	
**Response to Reviewers:**	Response: Thank you again for the comment. Sorry for our previous misunderstanding of pixel details. We have collected the pixel size details of each image and supplied in the online Excel, which can be download from

Powered by Editorial Manager® and ProduXion Manager® from Aries Systems Corporation

	(https://data.mendeley.com/datasets/xvs55d4rcz/draft?a=6223e44e-04b8-4705-91d9-bf98665c6194). According to the scale bars of the raw images, the average width or height pixel size of our multi-stage malaria images is in 11.842 μm to 18.947 μm.<br><br>The deep learning model has excellent feature learning capability by reasoning their representative class information from large amounts of different images with huge variations in size, shape or pixel values [1,2,3]. The pixel values in images must be normalized prior to providing the images as input to a deep learning neural network model during the training or evaluation of the model [4]. In our study, a total of 79672 multi-stage parasitized and uninfected cell images were cropped from raw images according to the given boxing coordinates. Those cell images with different pixel values were resized to 128*128 pixels before feeding into the deep neural networks. That is because each layer of deep neural networks only accepts image inputs of certain pixel value. Therefore, the deep learning models can handle the multi-stage malaria images with different pixel values. Our previous response is misleading, which has been corrected to “As the network accepts image inputs of certain pixel value，the input images with different number of pixels have been resized to 128*128 pixels before feeding into our deep learning model. ” According to your comments, we have added this description in Section 2.1 Data Acquisition (highlighting content).<br><br>[1] LeCun Y, Bengio Y, Hinton G. Deep learning[J]. nature, 2015, 521(7553): 436-444.<br>[2] Esteva A, Kuprel B, Novoa R A, et al. Dermatologist-level classification of skin cancer with deep neural networks[J]. nature, 2017, 542(7639): 115-118.<br>[3] Coudray N, Ocampo P S, Sakellaropoulos T, et al. Classification and mutation prediction from non–small cell lung cancer histopathology images using deep learning[J]. Nature medicine, 2018, 24(10): 1559-1567.<br>[4] Zheng X, Yao Z, Huang Y, et al. Deep learning radiomics can predict axillary lymph node status in early-stage breast cancer[J]. Nature communications, 2020, 11(1): 1-9.
**Additional Information:**	
**Question**	**Response**
Are you submitting this manuscript to a special series or article collection?	No
**Experimental design and statistics**<br><br>Full details of the experimental design and statistical methods used should be given in the Methods section, as detailed in our Minimum Standards Reporting Checklist. Information essential to interpreting the data presented should be made available in the figure legends.<br><br>Have you included all the information requested in your manuscript?	Yes
**Resources**<br><br>A description of all resources used, including antibodies, cell lines, animals and software tools, with enough information to allow them to be uniquely identified, should be included in the	Yes

Methods section. Authors are strongly encouraged to cite Research Resource Identifiers (RRIDs) for antibodies, model organisms and tools, where possible.<br><br>Have you included the information requested as detailed in our Minimum Standards Reporting Checklist?	
**Availability of data and materials**<br><br>All datasets and code on which the conclusions of the paper rely must be either included in your submission or deposited in publicly available repositories (where available and ethically appropriate), referencing such data using a unique identifier in the references and in the “Availability of Data and Materials” section of your manuscript.<br><br>Have you have met the above requirement as detailed in our Minimum Standards Reporting Checklist?	Yes

Manuscript Click here to access/download;Manuscript;DTGCN_s0321.pdf

Multi-Stage Malaria Parasites Recognition by Deep Learning

Sen Li[1], Zeyu Du[1], Xiangjie Meng[1], and Yang Zhang[1*]

[1]College of Science, Harbin Institute of Technology, Shenzhen, China.

*To whom correspondence should be addressed. Email: zhangyang07@hit.edu.cn

Abstract:

Motivation: Malaria, a mosquito-borne infectious disease affecting humans and other animals, is widespread in the tropical and subtropical regions. Microscopy is the most common method in diagnosing the malaria parasite from stained blood smears. However, the widespread manually under-microscope parasite examining is time-consuming, error-prone and well-trained-professional required. Besides, the recognition of a malaria parasite through microscope is still a challenging process, especially in distinguishing multiple stages of parasites.

Results: In this paper, we develop a novel deep learning approach for multi-stage malaria parasites recognition in blood smear images using Deep Transfer Graph Convolutional Network (DTGCN). And this paper is the first application of GCN on multi-stage malaria parasites recognition in blood smear images. The proposed DTGCN model is based on unsupervised learning by transferring knowledge learnt from source images that contain the discriminative morphology characteristics of multi-stage malaria parasites. These transferred information guarantees the effectiveness on the target parasites recognition. This approach firstly learns the identical representations from source to establish the topological correlations between source class centers and the unlabelled target samples. In this stage, the Graph Convolutional Network (GCN) is implemented to extract graph feature representations for the multi-stage malaria parasites recognition. The proposed method showed the higher accuracy and effectiveness in publicly available microscopic images of multi-stage malaria parasites compared to a wide range of state-of-the-art approaches. Furthermore, this method is also evaluated on a large-scale dataset of unseen malaria parasites and the *Babesia* dataset.

Availability: Code and dataset available at https://github.com/senli2018/DTGCN under an OSI compliant license (MIT).

Contact：*Email:zhangyang07@hit.edu.cn. Supplementary data are available online.

Keywords: *Malaria; Multi-stage recognition; Microscopic image analysis; Knowledge Transfer; Graph convolutional network; Deep learning.*

1 Introduction

The main source of Malaria is the parasites from the Plasmodium group which are transmitted to people though the bites of infected mosquitoes. People with malaria experience fever, chill, and a flu-like illness [1]. According to the summary from World Health Organization, there were 228 million cases of malaria worldwide, which resulted in approximately 405,000 deaths, in 2018. And there are approximately 94% of deaths occurred in African region [2]. Moreover, the risk of infectious disease (especially malaria) transmission is probably increased since the severe flooding inundated many regions in Asia this year [3]. This can make the malaria, one of the most serious public health problems, spread world widely. With the urgent situation, plenty of malaria relatively researches were conducted and the reported results showed that the hazard of malaria illnesses and deaths can be significantly reduced by accurate and affordable diagnostic testing, enabling better disease monitoring and control interventions.

Malaria is usually diagnosed by the microscopic examination of blood films, and hundreds of millions of blood films are examined every year for malaria diagnosis [2, 4]. Although this is the most widespread examination, the manually under-microscope examining is tedious and susceptibly leading error. Therefore, a considerable number of studies on computer-aided malaria detection systems have been proposed in the relevant literature [4, 5]. For example, the classification methods have been applied to discriminate between infected and uninfected red blood cells in thin smears or to identify parasites in thick smears, ranging from decision trees to basic artificial neural networks [6]. Furthermore, the recent studies have proven that the malaria diagnosis based on the deep learning architecture can significantly outperform the models based on conventional classifiers [7-10]. Liang et al. [7] applied a Convolutional Neural Network (CNN) approach to discriminate between infected and uninfected cells in thin blood smears, resulting in 97.37% accuracy on 27578 single cell images. And Dong et al. [8] evaluated three types of well-known CNNs, including LeNet, AlexNet, and GoogLeNet, which all achieved classification accuracies of over 95%. Gopakumar et al. [9] used CNN operating on a focus stack for automated quantitative detection of malaria parasites form blood smears with improved sensitivity (97.06%) and specificity (98.50%). Hung et al. [10] further developed a faster region-based CNN approach for object segmentation on malaria parasite images. The superior experimental results on 40,612 images segmentation demonstrate the effectiveness of the proposed model over the state-of-the-art method of traditional segmentation plus machine learning. This model also classified all objects for the segmented cells with finding that learning distinguishing features between infected classes is very challenging, which only achieved 59% accuracy. Thus, it turns to binary classification to identify the objects as red blood cell or not, with accuracy of 98%. Narayanan. et al [26]

proposed a fast CNN architecture and compared it with AlexNet, ResNet, VGG-16 and DenseNet models for the malaria detection. Findings showed that all tested methods achieved over 96% accuracies. Similar, Narayanan. et al [27] investigated the detection of malaria by using deep neural networks (GoogLeNet and ResNet), and obtained accuracies over than 96%.

Those methods mentioned above are particularly useful in detecting a single stage of malaria parasite, normally the ring form. However, the life cycle of the malaria parasites is quietly complicated. The entire cycle involves multiple morphological changes in human blood. As illustrated in Figure S1a, malaria parasites develop multi-stage forms with distinct microscopic presentations during its intraerythrocytic cycle, including gametocytes, rings, trophozoites, and schizonts [11]. Until now, an accurate multi-stage malaria detection system is still absent because of the morphological differences across multi-stage parasites and variations in images captured from different technicians, laboratories, clinics, and regions. Additionally, color variation, resulting from differences in staining pH, time, purity of dye, duration of the staining procedure and sensor settings (Figure S1b), is another challenge of multi-stage detection. All those morphological and hardware variations degrade the performance of models developed previously. Therefore, simply adaptation of existing single-stage classification models will perform poorly in multi-stage malaria parasites recognition. Another important problem is the lack of multi-stage-parasites training images with a balanced class distribution because of the dominance of ring stage parasites and RBCs captured under microscope.

To overcome the challenges in both variations and data imbalance for multi-stage malaria parasite recognition, we employ a transfer learning strategy by using the prior knowledge from the labeled source domain (existing scenario) to train the recognition model and apply it to an unlabeled target domain (even unseen scenario) for detection. On the other hand, the problem of data imbalance can be addressed by implementing Graph Convolutional Network (GCN) on the stablished topological correlations between source class groups and target features to bridge the different class distribution gaps. Specifically, GCNs have been proposed that whereby node features, aggregated from adjacent neighbors and different nodes, can share the same transfer function. Thus, the aggregated nodes can exploit more discriminative information according to the topological graph structure of the node features than directly utilizing CNN on a single image.

In this context, DTGCN is proposed for multi-stage malaria parasites recognition and classification, which consists of CNN-based feature extractor, a source transfer graph building component, and an unsupervised GCN. Based on the plenarily literature reviewing, none of the previously reported studies have attempted to explore the advantages of deep learning for multi-stage malaria parasite recognition. To demonstrate the effectiveness of proposed DTGCN, the research conducts experiments on two public malaria parasite image datasets, which are available from the Broad Bioimage Benchmark Collection [12] and the National Library of Medicine [13]. Furthermore, this proposed method is also evaluated on another parasite of *Babesia* data to show the robustness of DTGCN model. *Babesia* is a malaria-like parasite that infects red blood cells, and leads to the disease babesiosis [14]. With a ring like structure, the ring forms of *Babesia* are sometimes confused with those of the malaria parasites. The excellent result can prove that proposed DTGCN is not only limited to the malaria parasites recognition, but also effectively solve other microscopic image recognition problems. In general, the proposed DTGCN method can overcome the data variation and the imbalance problem in deep learning based malaria recognition. Importantly, this DTGCN method can transfer a sufficiently trained recognition model to a completely unlabeled target dataset with unknown differences (such as color, brightness or imaging settings).

2 Methods

2.1 Data Acquisition

The multi-stage malaria infected cell images are captured from blood smears stained with Giemsa reagent. This image set consists of totally 1364 images at 1000x magnification, and is publicly available at Broad Bioimage Benchmark Collection (BBBC) website (https://data.broadinstitute.org/bbbc/BBBC041/) [10]. All these images are manually captured from P. vivax infected patients in Manaus, Brazil, and Thailand under 1000X microscope, annotated by three different experts globally. This dataset contains images from two classes of uninfected cells (RBCs and leukocytes) and four classes of parasitized cells (gametocytes, rings, trophozoites, and schizonts) with Giemsa stained. Although the initial purpose of this dataset is used for the parasitized cell detection rather than multi-stage malaria parasites recognition, both bounding box coordinates and corresponding stage label were provided. A total of 79672 multi-stage parasitized and uninfected cell images were cropped from raw images according to the given boxing coordinates. As shown in Table S1, numbers of each class are severely imbalanced—97.2% of them are selected 5000 RBCs images. Since the Leukocyte class only contains 103 samples, this class is supplemented by self-captured 104 Leukocyte images for testing. With above data preparation, 100 images are randomly chosen from each class to form the testing dataset (600 samples in total) to evaluate the effectiveness of the proposed DTGCN. And the rest of images is employed as training data. To avoid the consumption of computing resources by the imbalance RBCs, 5000 random RBCs are selected in experiments to save training time, but achieve enough training efficiency. In total, this study uses 7456 microscopic images, containing 6856 images for training and 600 images for testing. The details of data distribution is shown in Table S1. In each image, there is only one parasite or cell. As the network accepts image inputs of certain pixel value, the input images with different number of pixels have been resized to 128*128 pixels before feeding into our deep learning model.

In addition, the second malaria parasite recognition task in this paper is classification of unseen malaria parasite in a large-scale dataset which has different distributions (such as brightness and imaging equipment settings) from source training dataset. The BBBC dataset is adapted as the source domain in this multi-stage malaria parasite recognition task, and it is transferred to two binary classes for recognition, which are parasitized class (gametocytes, rings, trophozoites, and schizonts) and uninfected class (RBCs and leukocytes). This dataset consists of 13780 testing images including both malaria parasites and red blood cells [13] and it is released at the website of National Library of Medicine (https://lhncbc.nlm.nih.gov/publication/pub9932). The images contain the segmented cells from Giemsa-stained thin blood smear slides of 150 P. falciparum-infected and 50 healthy patients under 1000X magnification. Moreover, another 1100 under-microscope *Babesia* and 1100 red blood cell images are collected to validate the generalizability of our proposed DTGCN model.

2.2 Framework of DTGCN

DTGCN is proposed for the multi-stage malaria parasites recognition, consisting of CNN feature learning, source transfer graph building, and

the Unsupervised Graph Convolutional Network (UGCN). Firstly, CNN is utilized to extract morphological features from images in each class. Secondly, a source transfer graph building algorithm is proposed to construct the class correlations between each source class groups and target samples by a proposed target-to-center source transfer graph building algorithm according to the source class labels. By transferring the representatively discriminative information from source into the target domain, it solves the challenges of variations for unseen scenario. Then, the CNN features and source transfer graph are together fed into graph convolutional layers, which is optimized by the UGCN loss. Finally, the network outputs the final graph representations with an unsupervised GCN loss preserving the identity information for the unlabeled target data. Given the transfer graph feature representations, we can conduct K-means clustering algorithm on the final target graph feature representations and achieve the multi-stage malaria parasite recognition in the target domain (Figure 1).

Figure 1. The schematic representation of DTGCN. Firstly, the CNN-based feature extractor is in charge of learning representations from source and target data, which is optimized by a fixed contrastive classification loss in the source domain, and a Maximum Mean Discrepancy (MMD) constraint for the feature-level transfer learning in the target domain. Next, the source transfer graph building component connects target sample features to the source class groups by a proposed target-to-center source transfer graph building algorithm to formulate the graph topology correlations. Finally, the unsupervised GCN learns the graph representations by feeding CNN features and the formulated source transfer graph.

2.3 CNN Feature Learning

As we all know, a crucial preliminary for image recognition is to extract representative morphological feature. For the multi-stage malaria parasite recognition task, we employ CNN as the backbone network due to its superiority performance in parasite microscopic image recognition [15], with excellent capability on dealing with challenging variations, such as illumination, background, and staining intensity. It can learn robust feature representations by multiplying overlapped convolutional operations with reasonable objective functions.

Mathematically, we assume the source data as $X_s = \{x_1^s, x_2^s, \cdots, x_{N_s}^s\}$ with complete labels $Y_s = \{y_1^s, y_2^s, \cdots, y_{N_s}^s\}$, and the target data as $X_t = \{x_1^t, x_2^t, \cdots, x_{N_t}^t\}$ without any labels which contains the same categories of source domain. To extract the appearance features by CNN, we define the backbone network as f_{cnn}, to learn the CNN representations for source and target images. In this paper, we utilize ResNet [16] architecture as the basic model of f_{cnn}. Then, given the i-th source image x_i^s and j-th target image x_j^t, the CNN features h_i^s and h_j^t can be calculated by,

$$h_i^s = f_{cnn}(x_i^s; \theta_{cnn}) \quad (1)$$
$$h_j^t = f_{cnn}(x_j^t; \theta_{cnn}) \quad (2)$$

where $h_i^s \in \mathbb{R}^d$, $h_j^t \in \mathbb{R}^d$ (d is the dimension of feature vectors), and θ_{cnn} is the learnable parameters in the backbone network f_{cnn}. These features are the vectors of pre-softmax units, that attaches an unsupervised graph convolutional network instead of prediction layers.

Importantly, to guarantee the CNN features can obtain the identity information for source images, source feature vectors are constrained by a contrastive loss function, denoted as CNN loss,

$$L_{cnn} = \frac{1}{2N_s} \sum_{i=1}^{N_s} ld^2 + (1-l)\max(m-d,0)^2 \quad (3)$$

where $d = \|h_i^s - h_k^s\|_2$ represents the Euclidean distance between two source features, $l = 1$ when $y_i^s == y_k^s$, otherwise $l = 0$, and m is the margin setting among the distances. The contrastive loss is to maintain the representative information cross different categories by learning a distance metric.

By this constraint, the backbone network f_{cnn} can learn discriminative feature representations only for source images. The left assignment with major significance of this part is how to transfer the learnt knowledge into target domain to bridge variations in the target scenario. To overcome this problem, a widely used strategy, Maximum Mean Discrepancy (MMD), is introduced to constrain the learnt source and target features,

$$L_{mmd} = \| \frac{1}{N_s} \sum_{i=1}^{N_s} h_i^s - \frac{1}{N_t} \sum_{j=1}^{N_t} h_j^t \|^2 \quad (4)$$

To further conduct the transfer learning and solve the problem of data imbalance, this paper proposes an unsupervised GCN.

2.4 Source Transfer Graph Building

Generally, GCN provides an effective solution to simulate the correlations between objects in different distributions [17]. Thus, this paper applies the transfer learning on GCN to alleviate the distribution-gap between source and target domains, and leverage the imbalance data in the source domain to exploit the topological structure in the feature space. GCN can exploit the multi-stage malaria infected cells by forwarding the message according to the node correlations based adjacent matrix, which is one of the most important steps in GCN. This section aims to formulate the topological correlation graph as the base of the graph convolution layers.

Considering the imbalance data in source domain, the network transfers the class center which contains most representative information by average pooling of class features. it will be recalled that there exists inherent correlations between the target images and the class centers in source domain because they belong to the same classes. Inspired by this point, a source transfer graph building mechanism is designed by introducing the source class centers into target domain.

By the CNN feature learning, the source and target image features ($H_s = \{h_1^s, h_2^s, \cdots, h_{N_s}^s\}$, $H_t = \{h_1^t, h_2^t, \cdots, h_{N_t}^t\}$) are obtained by Eq. 1 and 2 respectively. For the features of the source images, the class centers $\{h_c^n \in \mathbb{R}^d|_{n=1}^{N_c}\}$ are calculated for each class by equation below,

$$h_c^n = \frac{1}{\sum\limits_{y_i^s = n}} \sum_{i=1}^{N_s} (y_i^s == n) h_s^i \quad (5)$$

After above computing, the network can obtain N_c class centers of $H_c = \{h_c^1, h_c^2, \cdots, h_c^n, \cdots, h_c^{N_c}\}$ for source image features, which are deployed into the graph construction. Meanwhile, this model implements K-means clustering on target feature vectors H_t. Assume an adjacent matrix $A \in \mathbb{R}^{N_s \times N_t}$ representing the correlations between the source and target samples. This framework employs the distance of

target sample features to the source class center as the connected metrics. Specifically, given a target feature h_j^t, it is connected to the source class group when it has the smallest distance among all the source class centers,

$$\boldsymbol{A}_{kj} = \begin{cases} 0, & \text{if } \arg\min_n d(h_t^j, h_c^n) \neq y_k^s \ (1 \leq n \leq N_c) \\ 1, & \text{if } \arg\min_n d(h_t^j, h_c^n) == y_k^s \ (1 \leq n \leq N_c) \end{cases} \quad (6)$$

where $d(\cdot)$ is the Euclidean distance between target feature h_t^j and source class center h_c^n. This formulation ensures each target feature is connected with the nearest class group, and the framework can obtain the source transfer graph without any overlapped samples and connecting each other, as illustrated in Figure 1.

Compared with other existing graph building method, the most significant difference is the first graph building. The traditional methods create the first graph based on the CNN features, while, the proposed method employs the features to iteratively formulate the new graph within each epoch. The reason of this setting is that the learnt CNN feature provides discriminative information to the GCN, overcoming the imbalance problem in the image data.

2.5 Unsupervised Graph Convolutional Network

To strengthen the transfer learning ability of our model, we use graph convolutional network to extract representation of each target feature and group source class centers in an unsupervised manner. It consists a graph convolution stage and an unsupervised clustering objective function, which is in charge of malaria parasite recognition without any target annotation.

Given the source transfer graph in this paper, the complete graph $G(V, A)$ can be formulated, where $V = \{v_1, v_2, \cdots, v_n\}$ denotes the collection of the nodes with $|V| = N_t + N_c$, and $A \in \mathbb{R}^{N_c \times N_t}$ is the source transfer graph. Importantly, each node in this paper contains a feature vector from the CNN backbone f_{cnn}, where the nodes can be replaced by an integrated feature set H_i of $H_i = \{h_1^c, h_2^c, \cdots, h_{N_c}^c; h_1^t, h_2^t, \cdots, h_{N_t}^t\}$, which is composed by N_c source class center vector and N_t target feature vectors. Thus, the graph can be re-defined by $G(H_i, A)$. Since the GCN applied in semi-supervised framework [17] has achieved a series of successes, the graph convolutional layers are optimized by the classification cross-entropy loss, which needs several labelled samples. However, the target data without any labels cannot work. Hence, the UGCN is proposed on the graph G without utilizing any target labels. The primary components in UGCN is graph convolution layers in Figure 1,

$$H_g^{(l)} = f_{gcn}(H_g^{(l-1)}, \tilde{A}) = \sigma(\tilde{D}^{-1/2} \tilde{A} \tilde{D}^{-1/2} H^{(l-1)} W^{(l)}) \quad (7)$$

where $l = 1, 2, \cdots, L$ denotes the l-th graph convolution layer and L is the number of layers, $H^{(l-1)}$ and $H^{(l)}$ is the input and output graph features for l -th layer. Besides, $\tilde{A}$ represents the symmetrically normalized adjacent matrix with self-connections $(A + I)$, where I denotes the identity matrix. $\tilde{D}$ is the diagonal matrix of $\tilde{A}$, and W^l denotes the trainable weight parameters in l-th layer, and σ is the non-linear activation, which is ReLU function in this paper. According to the former GCN works [18], the deeper GCN with multiple layers may be harmful to the graph feature learning. Therefore, this paper also employs two graph convolutional layers to represent the final GCN features for each node, and the integrated CNN features are evolved as $H_g = \{h_1^g, h_2^g, \cdots, h_{N_c}^g; h_{N_c+1}^g, h_{N_c+2}^g, \cdots, h_{N_c+N_t}^g\}$. The final representations for malaria parasite images are obtained by graph convolutions on K-means-based graphs, showing more advantages in clustering than other clustering algorithms. To guarantee the unsupervised feature extraction of GCN on the target features, the K-means clustering algorithm [19] is implemented to learn N_c clusters $\{C_1, \cdots, C_k, \cdots, C_{N_c}\}$ with N_c feature collections $\{S_1, \cdots, S_k, \cdots, S_{N_c}\}$, and constrain the learnt H_g by UGCN loss,

$$L_{ugcn} = \frac{1}{N_c + N_t} \sum_{i=1}^{N_c+N_t} \sum_{h_g^i \in S_k} \|h_i^g - C_k\|^2 \quad (8)$$

where C_k can be calculated by,

$$C_k = \frac{1}{|S_k|} \sum_{h_i^g \in S_k} h_i^g \quad (9)$$

It should be emphasized that K-means clustering algorithm has excellent capability in calculating cluster centers in Euclidean distance space, and it can aggregate similar features by their Euclidean metrics, which is often utilized to formulate K nearest neighbour graph structure. Here, we set the CNN and GCN features to be an equivalent dimensions for the uniformity in Euclidean space, and adopt K-means algorithm to implement our source transfer graph building and unsupervised GCN module due to this distance metric consistency. In detail, the updating of K-means and the optimization of the H_g will be conducted iteratively one by one along with the training epochs (Algorithm S1). Thus, this network can achieve a satisfactory K-means clustering on the learnt graph features after fully trained.

2.6 Network Training and Evaluation Metrics

We present the details of network training, evaluation metrics and compared models in the supplementary (S4).

To demonstrate the superior effectiveness of our DTGCN models, we choose three widely used deep learning networks: Visual Geometry Group Network (VggNet) [20], the GoogLe Inception V3 Network (GoogLeNet) [21], and the deep Residual Network (ResNet) [16] to be the contrasts. Besides, we adopt four recently proposed malaria parasite recognition methods [22,23,24,25] with fine-tuning in our experimental datasets to conduct efficient comparison, and the proposed DTGCN is also modified to evaluate the core components of feature learning, graph building algorithm, and the unsupervised graph convolutional network. The modifications build two updated models: 1) removing the GCN by directly attaching graph features to K-means algorithm on the target CNN features (denoted as Baseline), and 2) using common KNN algorithm to formulate the graph (Ours+KNN). On the other hand, this study also evaluates the influence of the deeper CNN in feature learning, which is explored by changing the depth of ResNet: ResNet18, ResNet34 and ResNet50 are tested individually. Thus, there are two more models (Ours+ResNet34 and Ours+ResNet50) are evaluated.

3 Results

3.1 Performance on Multi-Stage Malaria Parasites Recognition

To validate the effectiveness of the DTGCN, this study first implements extensive experiments on multi-stage malaria parasite recognition. Several various models (VggNet, GoogLeNet, ResNet, Quinn et al [22], Rajaraman et al [23], Vijayalakshmi et al [24], Umer et al [25] and

DTGCN) are trained and evaluated in this section, and their performance are shown first five lines of upper part in Table 1. As for the baselines of recent papers, Quinn et al [22] designed a deep learning model trained from the annotated cell images with four hidden layers consisting of two convolution layers, one pooling layer and a fully connected layer. The performance of the deep neural networks was evaluated on the detection of malaria parasite in thick blood smears with an average precision of 97%. Rajaraman et al [23] employed pre-trained CNN based deep learning models as feature extractors to classify parasitized and uninfected cells, obtaining 95.7% classification accuracy on single-stage malaria detection. Vijayalakshmi et al [24] developed a novel transfer learning approach to identify infected malaria parasite, which is powered by combining VggNet and support vector machine. The results on malaria digital corpus images have achieved classification accuracy of 93.1%. Umer et al [25] applied pre-processing steps for re-sampling and normalizing input microscopy images and then utilized stacked CNN by fine-tuning it along with max-pooling and dropout layer. The performance of this model was evaluated on single stage malaria parasite detection, resulting in 99.98% accuracy. Specifically, these baseline methods are re-trained on the BBBC dataset and tested on the multi-stage malaria parasite images. From the results, the proposed DTGCN achieves an excellent performance with overall accuracy of 98.4%, and all of the Precision, Recall and F1-score are over 98%. Comparing with other CNN-used models, the proposed DTGCN has absolutely superior performance, because the best of other three models (GoogLeNet) only realized around 83% accuracy in the same task. Compared to DTGCN, recent works are originally proposed for single stage malaria parasite recognition, which is more vulnerable to the variations in multi-stage malaria parasites recognition. The variations degrade the performance of models developed previously, and has only achieved a maximum recognition accuracy of 66.3%. Therefore, simply adaptation of existing single-stage classification models into multi-stage malaria parasites recognition will perform poorly. In practical scene, the small scale of non-RBC images often occurs caused by the complicated image capturing operations, which is one of the most challenging problems in deep learning applications because deep learning requires large amount of data. In this paper, our model can train a robust feature learning network with only small number of non-RBC images, while exist deep learning methods cannot handle this challenging problem. Thus it reveals the excellent capability of our model on multi-stage malaria parasite recognition task.

Also, DTGCN classification of each stage of intra-erythrocytic cycle of malaria are also reported in lower part of Table1. For single stage classification, this model has the best performance in Gametocyte stage classifying and all the indicator is over 99%. While, the classification of RBC and Ring stage is slightly worse than others but still around 98% in every matrix. This might as a result of the Gametocyte stage has more obvious and abundant features. But the cell has not shown many abnormal properties in RBC and Ring stage (the sample images can be checked in Figure S1).

Table 1. Performance on multi-stage malaria parasites recognition.

**Performance of Ours and Compared Methods**				
**Method**	**Accuracy**	**Precision**	**Recall**	**F1-score**
VggNet	77.3±0.53	81.0±0.44	77.3±0.28	76.8±0.23
GoogLeNet	82.7±0.31	85.9±0.29	82.7±0.25	83.0±0.18
ResNet	81.0±0.28	87.7±0.21	81.0±0.19	81.5±0.20
[22]	60.3±0.48	77.1±0.36	60.3±0.30	55.13±0.34
[23]	61.5±0.35	78.0±0.28	61.5±0.31	56.9±0.20
[24]	66.3±0.29	79.0±0.26	66.3±0.23	63.0±0.23
[25]	30.0±0.32	43.1±0.37	30.0±0.42	16.4±0.30
Baseline	80.9±0.42	86.3±0.42	81.4±0.10	81.9±0.18
Ours+KNN	38.3±0.39	35.2±35	38.3±0.40	35.6±0.35
Ours+Res50	69.7±0.23	70.36±0.18	69.7±0.19	63.1±0.15
Ours+Res34	84.8±0.20	86.4±0.17	84.8±0.21	85.9±0.13
**Ours+Res18**	**98.4±0.19**	**98.5±0.16**	**98.5±0.19**	**98.5±0.13**
**Performance of Ours+Res18 on Each Stage**				
Gametocyte	99.3±0.58	99.7±0.25	99.3±0.58	99.5±0.40
Leukocyte	99.7±0.58	98.0±1.06	99.7±0.58	98.8±0.79
RBC	98.0±1.0	97.0±0.97	98.0±0.7	97.5±0.84
Ring	97.0±1.0	98.6±1.27	97.0±1.0	97.8±0.20
Schizont	98.3±1.2	98.0±0.22	99.0±1.0	98.5±0.61
Trophozoite	98.0±1.0	99.8±0.28	98.0±1.0	98.9±0.90

Note**: Proposed DTGCN method with the best results are highlighted in bold.

Additionally, the 2D t-SNE plot is deployed to show the clustering performance in Figure 2 (h) to visualize the capacity of the models in distinguishing multi-stage malaria parasites and uninfected cells. The t-SNE can be used to visualize high-dimensional data 2D maintaining local structures. In t-SNE, pairs of points are given joint probabilities based on their distance and the Kullback-Leibler divergence between the probabilities is minimized [10]. The t-SNE plots of 7 models are shown in Figure 2. Generally, the difference between multi-stage malaria parasites infected and uninfected cells is kind of clear and easy to distinguish. Specifically, Figure 2 (h) shows that the proposed DTGCN can learn the features that has less intra-class distance and larger cross-class distance, which means the same-class samples are clustered and the margin between multiple stages and uninfected samples are far enough to be distinguished easily by the following classifying procedure. However, the other-models-learnt features has not enough cross-class distances. For example, the VGG model (Figure 2 (a)) has especially non-clear clusters and this model has the worst performance in classification.

Figure 2. t-SNE performance on malaria parasites recognition. The t-SNE plots of VggNet (a), GoogLeNet (b), ResNet (c), and Baseline (d) are compared to various DTGCN approaches, including replacing the graph building graph by KNN algorithm (Ours+KNN) (e) , replacing the CNN backbones of ResNet-34 (Ours+Res34) (f), ResNet-50 (Our+Res50) (g), and ResNet-18 (Ours+Res18) (h). t-SNE plots provide a method to evaluate and refine clustering of each class of sample images. Data points are coloured according to their categories. DTGCN achieves the best t-SNE plot performance compared to other baselines. The t-SNE plot shows that Ours+Res18 is the best discriminated.

To better reveal the recognition result on the 600 testing images, this study employs the confusion matrix to visualize the accuracy of the multi-stage malaria parasites recognition in Figure 3 (h). Confusion matrix reveals the variation in misclassification between each class, and each column of the matrix represents the predicted class, where the summation of the column is equal to the predicted image number in this class. Each row in this matrix denotes the true classes and the summation of them is the total number of the real images in this class. From Figure 3 (h), it can be observed that proposed DTGCN only misclassify six images, of which five concentrate on RBC because the variation of RBC is more complicated. To sum up, DTGCN identifies malaria parasites effectively in multiple stages, and the DTGCN would be especially useful for humans.

Figure 3. Confusion matrixes for the multi-stage malaria parasites classification. The confusion matrixes of VggNet (a), GoogLeNet (b), ResNet (c), and Baseline (d) are compared to various DTGCN approaches, including replacing the graph building graph by KNN algorithm (Ours+KNN) (e) , replacing the CNN backbones of ResNet-34 (Ours+Res34) (f), ResNet-50 (Our+Res50) (g), and ResNet-18 (Ours+Res18) (h). Confusion matrix reveals the variation in misclassification between the predicted and true labels. The diagonal cells correspond to samples are correctly classified. The off-diagonal cells correspond to incorrectly classified samples. It is easily to see that Ours+Res18 (DTGCN) presents the best classification results on confusion matrix.

3.2 Performance on a Large-Scale Dataset of Malaria Parasites Recognition

In order to further validate the DTGCN's performance on a larger malaria parasite dataset, the four trained models from section 3.2 are directly tested on a larger scale dataset which consists of 13780 testing images with equally amount of infected and uninfected images. And this task is binary classification. To keep the consistency of the classes, gametocyte, ring, schizont, and trophozoite stages are grouped as the parasitized cells, and Leukocyte and RBCs are used as uninfected cells to conduct experiment. All models trained on the multi-stage malaria parasite images are directly transferred and tested on the large-scale malaria recognition dataset without further training. As shown in Table 2, our DTGCN achieves around 98% in all of the accuracy, F1-score, Precision, and Recall, compared to VggNet (72.4%), GoogLeNet (74.4%), ResNet (72.3%), Quinn et al [22] (51.1%), Rajaraman et al [23] (67.1%), Vijayalakshmi et al [24] (66.0%), Umer et al [25] (61.9%). These methods keep consistency with our DTGCN on Jaeser dataset following transfer learning strategy, that is, we train them only on source dataset and directly apply the models on target datasets. And VggNet still has the relative worse performance which keep the consistence with the last experiment. In addition, there is a clear separation between infected and uninfected cells in the t-SNE plot Figure S2 (h). Contrarily, the VggNet has the most mixture t-SNE with the worst classification results. This illustrates that the separable features are benefit the classification and this is exactly the operation the proposed DTGCN complete. Importantly, DTGCN does not require any target image labels. This property efficiently solves the problem of the large-scale-labelled data lacking which is caused by the highly time consumption of sufficient under-microscope labelling, labor-intensive and well-trained professionals requiring. The corresponding confusion matrixes are also displayed in Figure S3.

This large-scale data based binary classification task demonstrates that DTGCN method not only has superior performance in recognition of multi-stage malaria parasites, but also works well in an unseen large-scale malaria dataset. This extraordinary capacity saves the expensive labelling work in biomedical image analysis, and provides a novel ideology for unlabelled biomedical image classification. This part shows that DTGCN outperforms other alternative methods on the unseen large dataset of malaria parasites and it further demonstrates the generalizability of DTGCN in malaria parasites recognition.

Table 2. Performance on the large-scale malaria parasite dataset.

Performance of Ours and Compared Methods				
**Method**	**Accuracy**	**Precision**	**Recall**	**F1-score**
VggNet	72.4±0.23	74.2±0.19	72.4±0.22	71.8±0.27
GoogLeNet	74.4±0.22	81.0±0.18	74.4±0.23	72.9±0.21
ResNet	72.3±0.16	81.6±0.18	72.2±0.18	70.1±0.17
[22]	51.1±0.28	55.0±0.25	51.19±0.29	39.2±0.23
[23]	67.1±0.31	67.6±0.29	37.1±0.33	66.8±0.30
[24]	66.0±0.27	33.9±0.21	66.0±0.24	65.3±0.25
[25]	61.9±0.31	64.8±0.28	61.9±0.29	43.8±0.22
Baseline	74.9±0.17	74.9±0.25	74.9±0.17	74.9±0.19
Ours+KNN	52.0±0.39	54.8±0.30	52.0±0.38	44.0±0.33
Ours+Res50	87.1±0.10	87.1±0.05	87.1±0.12	87.1±0.09
Ours+Res34	95.7±0.11	96.0±0.04	95.7±0.08	95.9±0.07
**Ours+Res18**	**98.0±0.06**	**98.1±0.01**	**98.0±0.06**	**98.0±0.03**
**Performance of Ours+Res18 on Each Class**				
Parasitized	96.1±0.08	100.0±0.06	96.1±0.08	98.0±0.07
Uninfected	100.0±0.05	96.3±0.03	100.0±0.05	98.1±0.02

Note**: Proposed DTGCN method with best results are highlighted in bold.

3.3 Performance on Babesia Parasites Recognition

As mentioned in introduction, *Babesia* infects red blood cells and leads to the disease babesiosis. Since the clinical and laboratory presentations of babesiosis and malaria are quite similar (ring-like structure), the ring forms of *Babesia* are sometimes confused with those of the malaria parasite. Thus, a *Babesia* parasites recognition test is conducted in this study to distinguish between *Babesia* parasites and RBCs to evaluate the discriminant ability of proposed model for other parasites. With the similar ring shapes within infected red blood cells, the recognition task is also challenging for transfer learning. It is surprised that the proposed DTGCN can achieve 98.90% accuracy, 98.90% F1-score, 98.90% Recall and 98.92% Precision when training the model on 600 *Babesia* and 600 red blood cell images. The reason that this model can distinguish *Babesia* parasite and RBC might be the fact that DTGCN can successfully overcome the problem of insufficient and imbalance training data. This experiment shows that proposed DTGCN is effective, flexible, and scalable when presented with challenging microscopic object recognition task.

3.4 Further Analysis of our DTGCN

The proposed DTGCN for multi-stage malaria recognition is composed by three major modules of CNN feature learning, target-to-center source transfer graph building, and the unsupervised graph convolutional network. In this section, we will discuss each main component in this model about their capacity and try to explore the reasons why this collaborated model works excellently.

3.4.1 Analysis of CNN feature learning module

To analyse the contribution of CNN feature learning, and evaluate the influence of deeper CNNs, we extract CNN features by deeper ResNets of ResNet34 and ResNet50 rather than ResNet18. The relative recognition results are summarized in Table 1 and 2 for multi-stage malaria parasites and large scale binary malaria parasites images, respectively, which t-SNE and confusion matrixes is also illustrated in Figure 2-3, and Figure S2-S3, respectively. Generally, the deeper network usually provides better feature learning ability for image classification, which benefits from the large scale and class-balance data (e.g. ImageNet). However, DTGCN achieves the best accuracy when the model employs ResNet-18, rather than the deeper networks of ResNet-34 and ResNet-50 as the CNN feature extractor. Taking the multi-stage malaria parasites recognition as an example, DTGCN(Ours+Res18)

obtains 98.4% accuracy, while Ours+Res34/50 achieves 84.8%/69.7%. It is easily to see that the shallow CNN (e.g. ResNet18) generates the highest accuracy and deeper CNN achieves weaker performance. The reason why this phenomenon is the small data volume and class-imbalance in source domain limits the deeper CNNs to a well-training for multi-stage malaria parasites recognition. In another word, the available multi-stage malaria parasites images can not satisfy the requirements of deeper ResNets with 34 and 50 layers, and this paper contrarily utilizes the shallow CNN of ResNet-18 to preserve more appearance features and the proposed UGCN module overcomes the problem of imbalance and insufficient training data.

3.4.2 Analysis of source transfer graph building module

As the major step of GCN, graph building algorithm is extremely important to establish a natural and topological structure for GCN. This paper involves a novel graph building mechanism, named as target-to-center source transfer graph building algorithm, which connects the target image features to the source class centers by measuring the distance between the sample's cluster to source centers.

To show the contribution of proposed graph building algorithm for multi-stage malaria parasites recognition, we deploy a well-known KNN graph building algorithm, which is usually employed in GCN, to replace our module. This modified method is named by Ours+KNN in Table 1 and 2, which only produces accuracies of 38.3% and 52.0% for multi-stage and binary malaria parasites recognition. The absolute superiority of the performance on t-SNE and confusion matrixes in Figure 2-3, and Figure S2-S3 of the proposed source transfer graph building algorithm proves that the established GCN are a reasonable and robust topological method for solving the transfer learning problem in microscopic image recognition.

3.4.3 Analysis of unsupervised graph convolutional network

The crucial module of DTGCN is the proposed unsupervised GCN, which aims to explore the correlations between the unlabelled target samples given the inputs of CNN features and graph. To comprehensively evaluate the contribution of UGCN, we remove this module from DTGCN and directly utilize K-means algorithm on the target CNN features which is extracted from the sufficiently trained CNN feature learning module. This modification is defined as the Baseline of proposed DTGCN, and the detail results on BBBC datasets are reported in Table 1. From the comparison of DTGCN and Baseline, the UGCN improves the accuracy of 17.5% (98.4-80.9), as well as large increasing in other metrics. From that, the proposed DTGCN appears an overall preponderance over Baseline, because of the contribution of UGCN. To further demonstrate the benefit from UGCN, this paper also visualizes the t-SNE of Baseline on multi-stage malaria parasites recognition (Figure 2 d) and large-scale malaria parasite (Figure S2 d), as well as confusion matrixes (Figure 3 d and Figure S3 d). In terms of the recognition results and the visualization comparison, the significant contribution of UGCN on exploiting the discriminant topological correlations is fully presented and proved.

3.4.4 Influence of the training data size (in source domain)

In general transfer learning tasks, the source data plays an important role in the domain adaptation, and the efficacy of the transferred model is most relied on the scale of source data. To evaluate the influence of training data size in our DTGCN, different data sizes from the source domain are randomly selected from each class with equal percentages of [20%, 40%, 60%, 80%, 100%] to conduct transfer learning on the target multi-stage malaria parasites dataset. As shown in Figure S4, box plot is introduced to visualize the accuracy results by conducting five randomly repeated times. Observing the plot, DTGCN realizes around 78 % accuracy when utilizing 20% and 40% source data. Furthermore, DTGCN achieves an acceptable accuracy of 89.93% when employ 60% of source data, instead of using entire source dataset. This demonstrates that proposed DTGCN is trainable with limited data. This analysis concludes that the more source training data, the better transfer learning performance will be achieved. And this result suggests the researchers should utilize as much source data as possible to support the transfer learning on target domain.

4 Discussion and Conclusion

This study is the first to investigate multi-stage malaria parasite recognition with the use of a Deep Transfer Graph Convolutional Network (DTGCN) approach. In this paper, the DTGCN consists of three major components: CNN for feature learning, source transfer graph building, and unsupervised GCN.

We design the source transfer graph building and unsupervised graph convolutional network for multi-stage parasites recognition, aiming to solve the problems of data variations and imbalance problem. Knowing this, the transfer learning between labelled source data to unlabelled target data can work in many scenarios. The proposed model firstly learns the CNN features by a ResNet architecture with constraints of MMD and Contrastive losses. Then utilizes the target-to-center based source transfer graph building algorithm to connect the source class centers with target samples, to leverage the imbalance data. After CNN feature learning and graph building, DTGCN employs unsupervised graph convolutional network to further alleviate the feature distribution gap between source and target domains by a K-means supervision. Thus, the proposed framework can achieve the multi-stage malaria parasites recognition results by the K-means algorithm on the final graph feature representations from target domain. What needs to be emphasized is that proposed DTGCN not only works out the supervised multi-stage malaria parasites recognition task, but also does not require any microscopic image labels in the target domain, which means that this model can be transferred to solve an unseen scenario to conduct recognition. Proposed DTGCN can also be applied to other biomedical image recognition tasks which have complicated procedures for data collection and annotation.

Through experiments on publicly available multi-stage and binary microscopic malaria parasite images, this paper has successfully demonstrated that DTGCN model can extract information to boost the accuracy of deep learning. Results on malaria-like parasites *Babesia* show that DTGCN model can be used for detecting other parasites under microscope.

The proposed method for multi-stage malaria parasite microscopic image analysis can be immensely helpful in the development of a low-cost, automated malaria diagnostic solution. This can significantly improve efficiency and reduce the need for dedicated pathologists in areas with limited resources.

Funding

The project is financially supported by the Natural Science Foundation of Shenzhen City (Project number JCYJ20180306172131515).

Declaration of interest

The authors declare that the research was conducted in the absence of any commercial or financial relationships that could be construed as a potential conflict of interest.

Author contributions

Y. Z contributed to the supervision of the study. Y. Z, and S. L contributed to the design of the study. S. L, Z. D and X. M performed data. All authors contributed to literature review, writing, and critical revision of the manuscript.

Availability of supporting source code and requirements

Project name: Deep Transfer Graph Convolutional Network

Project home page: https://github.com/senli2018/DTGCN

Operating system(s): Windows 10 with Nvidia Geforce 2080Ti GPU

Programming language: Python 3.6.0

Other requirements: Pytorch 1.0.0 or higher, Torchvision 0.4.1 or higher, Scipy 1.1.0 or higher, and Numpy 1.17.4 or higher.

License: MIT License (an OSI compliant license)

RRID: SCR_020976

Biotools: deep_transfer_graph_convolutional_network

References

[1]. Law, Y.J.N., *Rare human outbreak of monkey malaria detected in Malaysia.* Nature, 2018. **16**.

[2]. WHO. *World malaria report 2019*. 2019 [cited 20120 June, 29]; Available from: https://www.who.int/news-room/feature-stories/detail/world-malaria-report-2019.

[3]. Okaka, F.O. and B.D.O. Odhiambo, *Relationship between Flooding and Out Break of Infectious Diseasesin Kenya: A Review of the Literature.* Journal of Environmental and Public Health, 2018. **2018**: p. 1-8.

[4]. Poostchi, M., et al., *Image analysis and machine learning for detecting malaria.* Translational Research, 2018. **194**: p. 36-55.

[5]. Das, D.K., R. Mukherjee, and C. Chakraborty, *Computational microscopic imaging for malaria parasite detection: a systematic review.* Journal of microscopy, 2015. **260**(1): p. 1-19.

[6]. Jan, Z., et al., *A review on automated diagnosis of malaria parasite in microscopic blood smears images.* Multimedia Tools and Applications, 2018. **77**(8): p. 9801-9826.

[7]. Liang, Z., et al. *CNN-based image analysis for malaria diagnosis*. in *2016 IEEE International Conference on Bioinformatics and Biomedicine (BIBM)*. 2016. IEEE.

[8]. Dong, Y., et al. *Evaluations of deep convolutional neural networks for automatic identification of malaria infected cells*. in *2017 IEEE EMBS International Conference on Biomedical & Health Informatics (BHI)*. 2017. IEEE.

[9]. Gopakumar, G., et al., *CNN based malaria diagnosis from focus-stack of blood smear images acquired using custom-built slide scanner.* Online Wiley Library, 2018.

[10]. Hung, J. and A. Carpenter. *Applying faster R-CNN for object detection on malaria images*. in *Proceedings of the IEEE conference on computer vision and pattern recognition workshops*. 2017.

[11]. Phillips, M., et al., *Malaria. Nature reviews.* Disease primers, 2017. **3**.

[12]. Ljosa, V., K.L. Sokolnicki, and A.E.J.N.m. Carpenter, *Annotated high-throughput microscopy image sets for validation.* 2012. **9**(7): p. 637-637.

[13].Sivaramakrishnan, R., et al., *Pre-trained convolutional neural networks as feature extractors toward improved malaria parasite detection in thin blood smear images.* Peerj, 2018. **6**: p. e4568-.

[14].Homer, M.J., et al., *Babesiosis.* Clinical microbiology reviews, 2000. **13**(3): p. 451-469.

[15]. Li, S., et al., *Transfer Learning for Toxoplasma gondii Recognition.* Msystems, 2020. **5**(1).

[16]. He, K., et al. *Deep residual learning for image recognition*. in *Proceedings of the IEEE conference on computer vision and pattern recognition*. 2016.

[17].Kipf, T.N. and M.J.a.p.a. Welling, *Semi-supervised classification with graph convolutional networks.* 2016.

[18].Liu, S., et al., *Higher-order Weighted Graph Convolutional Networks.* arXiv preprint arXiv:1911.04129, 2019.

[19].Jain, A.K., *Data clustering: 50 years beyond K-means.* Pattern recognition letters, 2010. **31**(8): p. 651-666.

[20]. Simonyan, K. and A. Zisserman. *Very deep convolutional networks for large-scale image recognition*. in IEEE *computer vision and pattern recognition*. 2014.

[21]. Szegedy, C., et al. *Inception-v4, Inception-ResNet and the Impact of Residual Connections on Learning*. in *national conference on artificial intelligence*. 2016.

[22]. Quinn J A, Nakasi R, Mugagga P K B, et al. Deep convolutional neural networks for microscopy-based point of care diagnostics[C]//Machine Learning for Healthcare Conference. 2016: 271-281.

[23]. Rajaraman S, Antani S K, Poostchi M, et al. Pre-trained convolutional neural networks as feature extractors toward improved malaria parasite detection in thin blood smear images[J]. PeerJ, 2018, 6: e4568.

[24]. Vijayalakshmi A. Deep learning approach to detect malaria from microscopic images[J]. Multimedia Tools and Applications, 2020, 79(21): 15297-15317.

[25]. Umer M, Sadiq S, Ahmad M, et al. A Novel Stacked CNN for Malarial Parasite Detection in Thin Blood Smear Images[J]. IEEE Access, 2020, 8: 93782-93792.

[26]. Narayanan B N, Ali R, Hardie R C. Performance analysis of machine learning and deep learning architectures for malaria detection on cell images[C]//Applications of Machine Learning. International Society for Optics and Photonics, 2019, 11139: 111390W.

[27]. Narayanan B N, De Silva M S, Hardie R C, et al. Understanding deep neural network predictions for medical imaging applications[J]. arXiv preprint arXiv:1912.09621, 2019.

Figure 1

Click here to access/download;Figure;Figure1(1).pdf

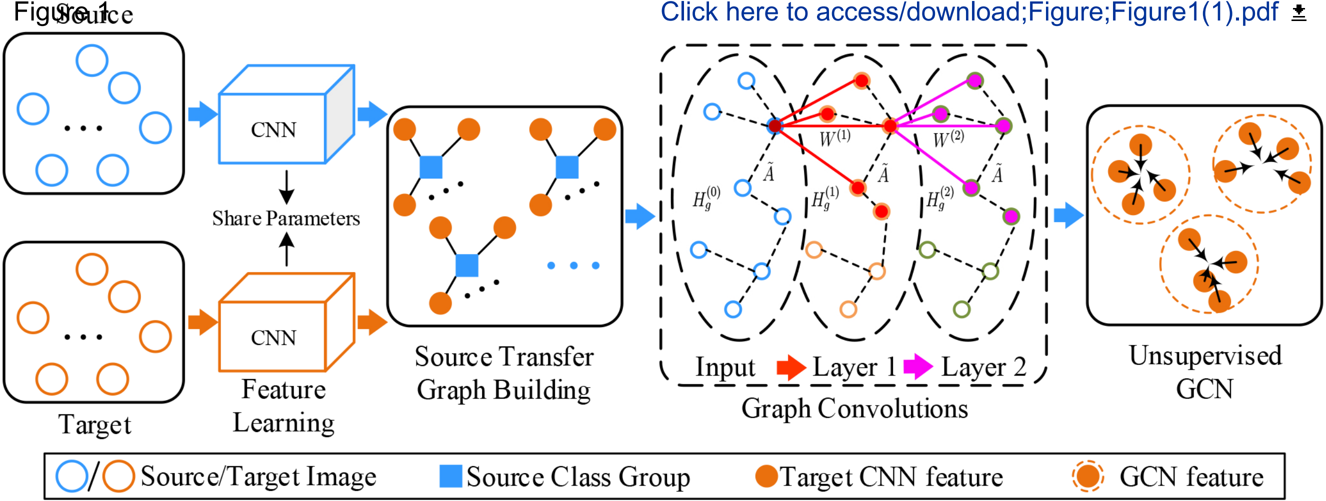

Figure 2

Click here to access/download;Figure;Figure2(2).pdf

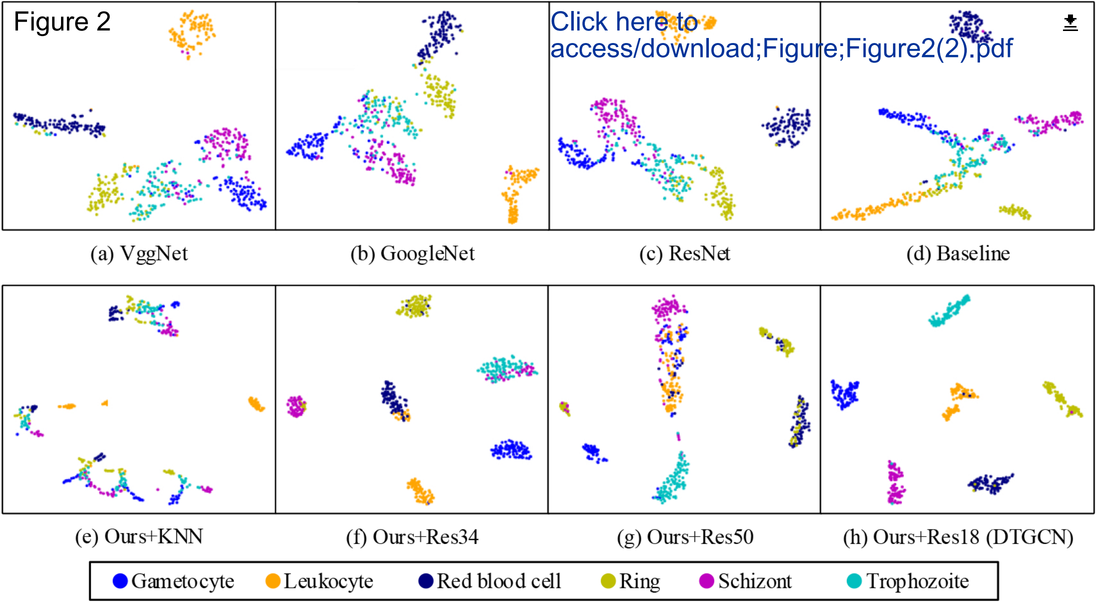

Figure 3

Click here to access/download;Figure;Figure3(1).pdf

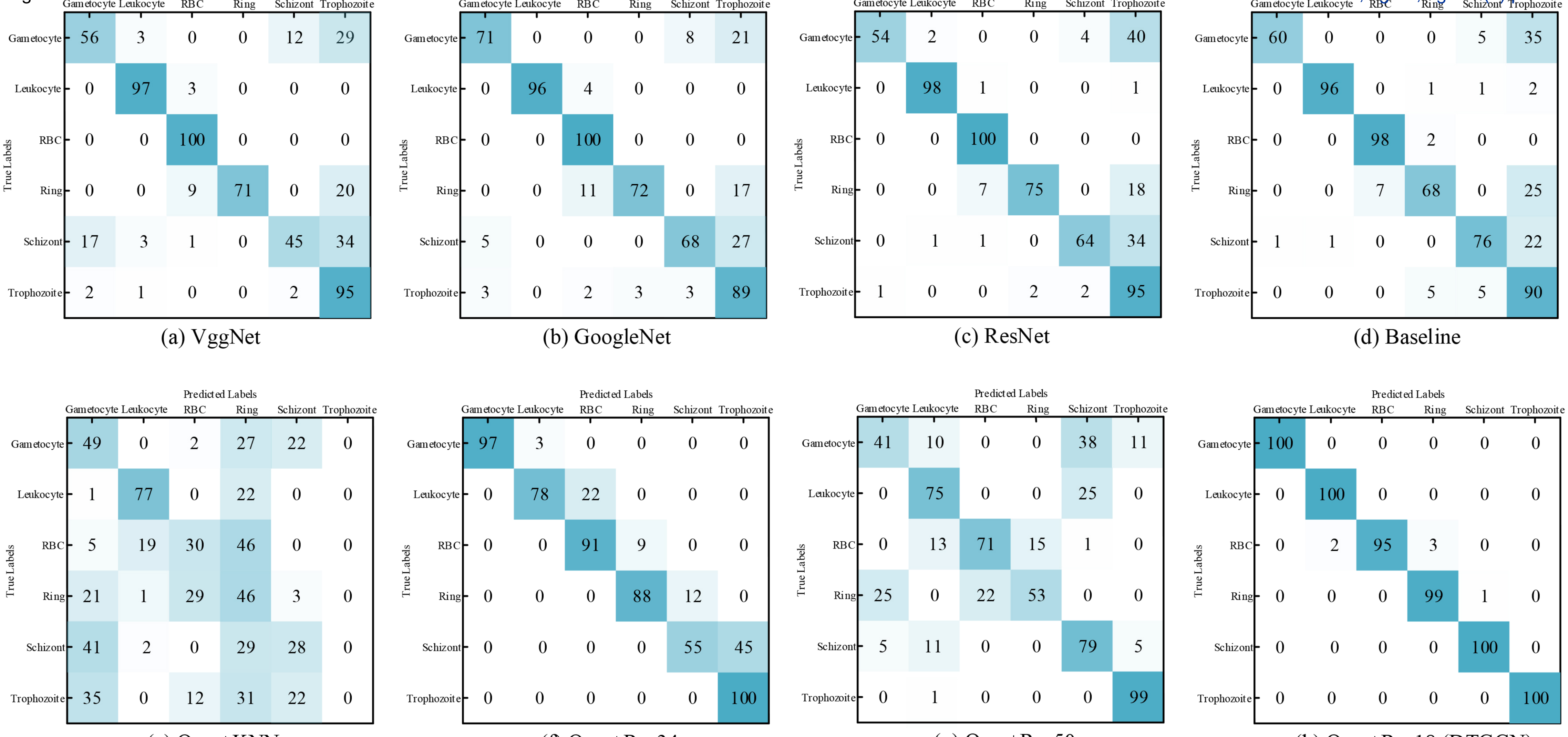

(a) VggNet (b) GoogleNet (c) ResNet (d) Baseline

(e) Ours+KNN (f) Ours+Res34 (g) Ours+Res50 (h) Ours+Res18 (DTGCN)

Click here to access/download
Supplementary Material
DTGCN_sup_bio0302.pdf

Personal Cover

Click here to access/download;Personal Cover;point-to-point response of gigascience_0320.doc

哈爾濱工業大學 HARBIN INSTITUTE OF TECHNOLOGY

School of Science
Harbin Institute of Technology
Shenzhen, Guangdong 518055
P.R. China

March 21, 2021

Dear Dr. Nicole,

Thank you for the review of our manuscript entitled “Multi-Stage Malaria Parasites Recognition by Deep Learning" (No. GIGA-D-21-00019R1). After fully considering the opinions of reviewers and making appropriate and complementary revision (marked in yellow) to the manuscript, we believe it will be of considerable interest to the broad readership of *GigaScience*. We are hereby resubmitting the revised manuscript, which has been carefully revised according to the reviewers' comments and suggestions. Point-by-point responses to the comments are appended, as well as the revised manuscript.

The authors appreciate your consideration of our manuscript again, and we look forward to hearing from you soon.

With my best regards

Yang Zhang
Email: zhangyang07@hit.edu.cn

Response to reviewers

Response to Reviewer #

Comments:

I would be more inclined to believe the validity of this approach if the authors were able to provide pixel size details (e.g. 9 micron x 9 micron) that we would enable me to make measurements of the spots in their image dataset. However, once again the authors have not provided the requested pixel size details. The Excel sheet the authors have provided does not provide pixel size details. To illustrate with one example from the Excel sheet, consider image [a1016_81] where the authors provide the following details: pixel width=194, pix el height=93. Presumably, these refer to the number of pixels in an image. However, to calculate the dimensio ns - or area - of the various spots that are the features used by the authors to determine the stage of the mala ria parasites, I would need to know the pixel size. By pixel size, I mean the dimensions - in microns - of one s quare pixel. As this image dataset is highly variable, I require these details for each image in the imaging dat aset. Without these details, measurements cannot be made on these image data, and the resizing argument us ed by the authors in their response - "Although these image pixels are different, they have been resized into t he same pixel setting by the pre-processing process in the deep learning model" - is quite frankly, absurd.

For a third time, I invite the authors to provide pixel size details for each of the images used in their analysis. Pixel size details will enable me to make measurements of the features that the authors use in their automate d feature detection analysis. By assessing these measurements, I wish to determine whether the computationa l analysis outlined in the manuscript is valid.

Response: Thank you again for the comment. Sorry for our previous misunderstanding of pixel details. We have collected the pixel size details of each image and supplied in the online Excel, which can be download from (https://data.mendeley.com/datasets/xvs55d4rcz/draft?a=6223e44e-04b8-4705-91d9-bf98665c6194). According to the scale bars of the raw images, the average width or height pixel size of our multi-stage malaria images is in 11.842 μm to 18.947 μm.

The deep learning model has excellent feature learning capability by reasoning their representative class information from large amounts of different images with huge variations in size, shape or pixel values [1,2,3]. The pixel values in images must be normalized prior to providing the images as input to a deep learning neural network model during the training or evaluation of the model [4]. In our study, a total of 79672 multi-stage parasitized and uninfected cell images were cropped from raw images according to the given boxing coordinates. Those cell images with different pixel values were resized to 128*128 pixels before feeding into the deep neural networks. That is because each layer of deep neural networks only accepts image inputs of certain pixel value. Therefore, the deep learning models can handle the multi-stage malaria images with different pixel values. Our previous response

is misleading, which has been corrected to “As the network accepts image inputs of certain pixel value，the input images with different number of pixels have been resized to 128*128 pixels before feeding into our deep learning model. ” **According to your comments, we have added this description in Section 2.1 Data Acquisition (highlighting content).**

[1] LeCun Y, Bengio Y, Hinton G. Deep learning[J]. nature, 2015, 521(7553): 436-444.

[2] Esteva A, Kuprel B, Novoa R A, et al. Dermatologist-level classification of skin cancer with deep neural networks[J]. nature, 2017, 542(7639): 115-118.

[3] Coudray N, Ocampo P S, Sakellaropoulos T, et al. Classification and mutation prediction from non–small cell lung cancer histopathology images using deep learning[J]. Nature medicine, 2018, 24(10): 1559-1567.

[4] Zheng X, Yao Z, Huang Y, et al. Deep learning radiomics can predict axillary lymph node status in early-stage breast cancer[J]. Nature communications, 2020, 11(1): 1-9.
