# Supplementary material for: Multi-stage malaria parasite recognition by deep learning
Source: Gigascience. 2021 Jun 17;10(6):giab040. doi: 10.1093/gigascience/giab040 (PMC8210472; doi:10.1093/gigascience/giab040)
Supplement: giab040_Supplemental_File [file giab040_supplemental_file.pdf]

# Supplementary

## 1. The life cycle of malaria parasites

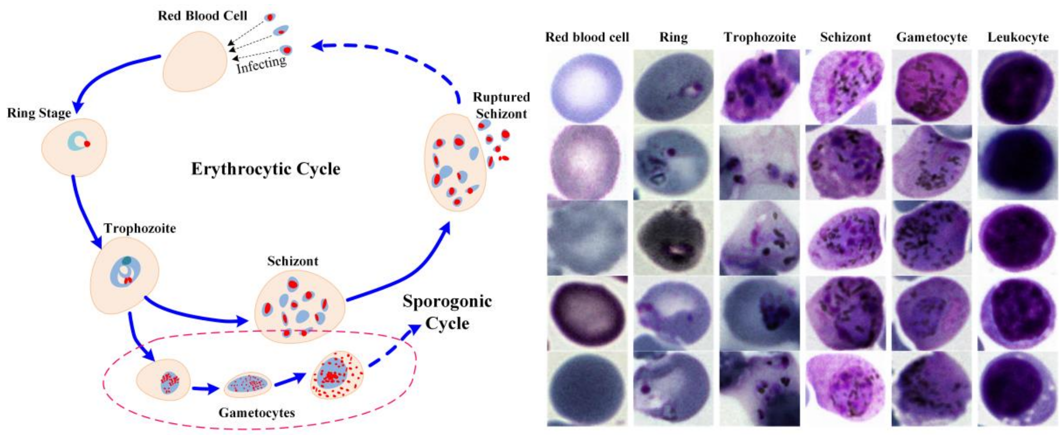


(a) intra-erythrocytic cycle (b) Malaria parasite image samples in each class

**Figure S1. The life cycle of malaria parasites. (a) The intra-erythrocytic cycle of malaria parasite; (b) Examples of multi-stage malaria parasites.** Malaria parasites undergo several stages in their complex lifecycle. The malaria parasites undergo repeated rounds of asexual multiplication (the intra-erythrocytic developmental cycle). During the intra-erythrocytic cycle, parasites go through the ring, trophozoite and schizont stages. In each cycle, a small proportion of parasites begin to develop into the sexual form of the parasite, which is known as a gametocyte.

## 2. The details of multi-stage malaria parasite images

**Table S1. The details of multi-stage malaria parasite images used in this study.** Training images of multi-stage parasites with imbalanced class distribution, where most images captured under microscope are red blood cells. This table illustrates the number of training and test data.

| Class | Training | Testing | Total |
|---|---|---|---|
| Gametocyte | 44 | 100 | 144 |
| Leukocyte | 107 | 100 | 207 |
| Red Blood Cell (RBC) | 5000 | 100 | 5100 |
| Ring | 253 | 100 | 353 |
| Schizont | 79 | 100 | 179 |
| Trophozoite | 1373 | 100 | 1473 |
| Total | 6856 | 600 | 7456 |

## 3. Algorithm of Deep Transfer Graph Convolutional Network

**Algorithm S1 Deep Transfer Graph Convolutional Network**

**Input:** Source data $X_s$, $Y_s$, and target data $X_t$; Parameters $m$, balance parameter $\lambda$; Batch size=15

**Initialization:** Initialize weight parameters in our network and learning rate.

**For** epoch=0 to $M$ **do**

  Sample mini-batch image pairs from source domain and sample mini-batch images from target domain.

  Updating the CNN feature learning module by minimizing Eq. 3 and Eq. 4.

**End For**

Source Transfer Graph Building according to Eq. 5 and Eq. 6.

**For** epoch=0 to $N$ do

  Updating the UGCN module by minimizing Eq. 8.

  Updating the cluster points by Eq. 9

**End for**

**Return parameters in DTGCN**

**Algorithm S1. Deep Transfer Graph Convolutional Network Algorithm.** This algorithm uses a DTGCN to alleviate the domain gap between the source and target domains to solve the class-imbalance problem. This algorithm employs $X_s$, $Y_s$ as input source data and $X_t$ as target data. Then, the network is optimized using losses of CNN and UGCN with the initialization of parameters in *M* iterations. Finally, the target images $X_t$ is tested by conducting *K*-means on the learned GCN features.

## 4. Network Training and Evaluation Metrics

As mentioned in former paragraphs, the CNN feature learning with ResNet50 architecture is optimized along with GCN. In this section, the batch size is set as 30/10 for source/target data with learning rate of 1e-5 which multiply 0.1 in every 50 epochs, the margin parameter $m = 5$ in Eq. (3) and the balance parameter $\lambda = 1$ for $L_{cnn}$ and $L_{mmd}$ to formulate the overall loss function $L = L_{cnn} + \lambda L_{mmd}$ of CNN. For the training details, the two graph convolution layers are trained with the output CNN features of the pre-trained CNN. For both of CNN and GCN training, the DTGCN is implemented by the PyTorch framework with GTX2080Ti GPU and employ Adam optimizer to optimize the parameters of network. The detailed setting is shown in Table S2. And the settings of large-scale malaria parasites recognition are summarized in Table S3.

**Table S2. Experimental settings of the compared methods on multi-stage malaria parasites recognition.** Details regarding epoch number, batch size for source/target data, learning rate, and optimizer were summarized. The learning rates of each method are initialized with the value in the Table S2, and will multiply 0.1 in each 50 epochs along with the training. In the modified methods of DTGCN, the maximum epoch number is 50, learning rate is 1e-5, and optimizer is Adam both for CNN feature learning and UGCN modules.

| Methods | Epoch Number | BatchSize | Learning Rate | Optimizer |
|---|---|---|---|---|
| VggNet | 50 | 70 | 1e-5 | Adam |
| GoogLeNet | 50 | 70 | 1e-5 | Adam |
| ResNet | 50 | 30 | 1e-5 | Adam |
| Baseline | 50 | 30 | 1e-5 | Adam |
| Ours+KNN | 50 | 30/10 | 1e-5 | Adam |
| Ours+Res18 | 50 | 30/10 | 1e-5 | Adam |
| Ours+Res34 | 50 | 30/10 | 1e-5 | Adam |
| Ours+Res50 | 50 | 30/10 | 1e-5 | Adam |

**Table S3. Experimental settings of the compared methods on large scale malaria parasites recognition.** Details regarding epoch number, batch size for source/target data, learning rate, and optimizer are summarized. The learning rates of each method are initialized with the value in the Table S3 and will multiply 0.1 in each 50 epochs along with the training. In the modified methods of DTGCN, the maximum epoch number is 50, learning rate is 1e-5, and optimizer is Adam both for CNN feature learning and UGCN modules.

| Methods | Epoch Number | BatchSize | Learning Rate | Optimizer |
|---|---|---|---|---|
| VggNet | 50 | 70 | 1e-5 | Adam |
| GoogLeNet | 50 | 70 | 1e-5 | Adam |
| ResNet | 50 | 30 | 1e-5 | Adam |
| Baseline | 50 | 30 | 1e-5 | Adam |
| Ours+KNN | 50 | 30/10 | 1e-5 | Adam |
| Ours+Res50 | 50 | 30/10 | 1e-5 | Adam |
| Ours+Res34 | 50 | 30/10 | 1e-5 | Adam |
| Ours+Res18 | 50 | 30/10 | 1e-5 | Adam |

To evaluate the overall performance of proposed DTGCN method, this paper reports the average recognition accuracy of parasites recognition, and computes the average Precision, Recall, and F1-score for evaluation. Moreover, T-SNE is visualized as plot [1] for extracted GCN features and draw the confusion matrix of the prediction statistics. The measurements of accuracy, precision, recall, and F1-score are originally designed for binary classification, their original definitions are described as blow:

$$Accuracy = \frac{TP+TN}{TP+TN+FP+FN} \quad (10)$$

$$Recall = \frac{TP}{TP+FN} \quad (11)$$

$$Precision = \frac{TP}{TP+FP} \quad (12)$$

$$F1\text{-}score = \frac{2*Recall*Precision}{Recall+Precision} \quad (13)$$

where $TP$ is the number of correctly predicted positive samples, $TN$ denotes the number of correctly predicted negative samples, $FP$ represents the number of false predicted positive samples, and $FN$ is the number of false predicted negative samples.

## 5. t-SNE Performance on a Large-Scale Dataset of Malaria Parasites Recognition

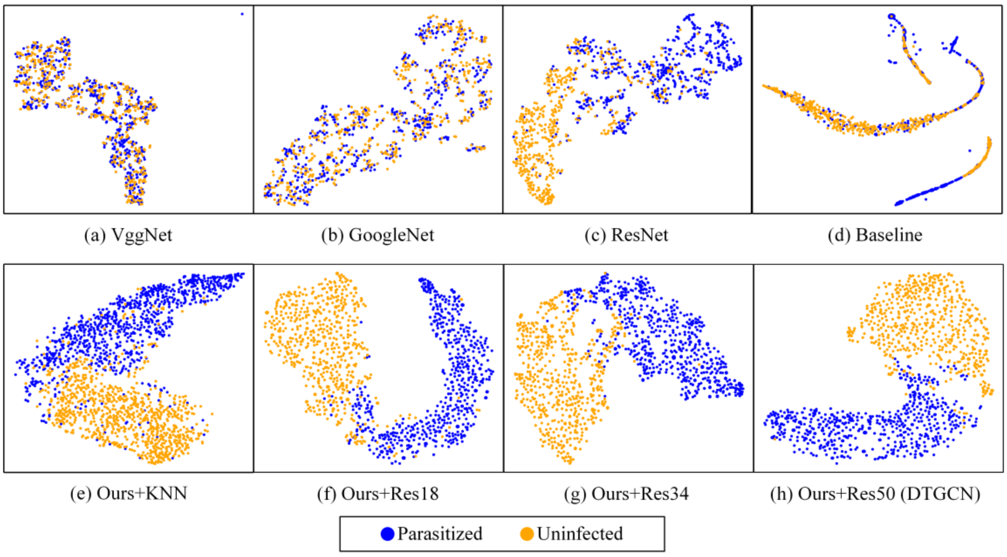


**Figure S2. t-SNE performance on large scale malaria parasites binary classification.** The t-SNE plots of VggNet (a), GoogleNet (b), ResNet (c), and Baseline (d) are compared to various DTGCN approaches, including replacing the graph building

graph by KNN algorithm (Ours+KNN) (e) , replacing the CNN backbones of ResNet-18 (Ours+Res18) (f), ResNet-34 (Our+Res34) (g), and original ResNet-50 (Ours+Res50) (h). The t-SNE plots provide a method to evaluate and refine clustering of each class of sample images. Data points are coloured according to their categories. The performance on large scale malaria parasites classification is similar to the multi-stage parasites classification, showing Ours+Res50 is the best discriminated.

## 6. Confusion Matrixes on a Large-Scale Dataset of Malaria Parasites Recognition

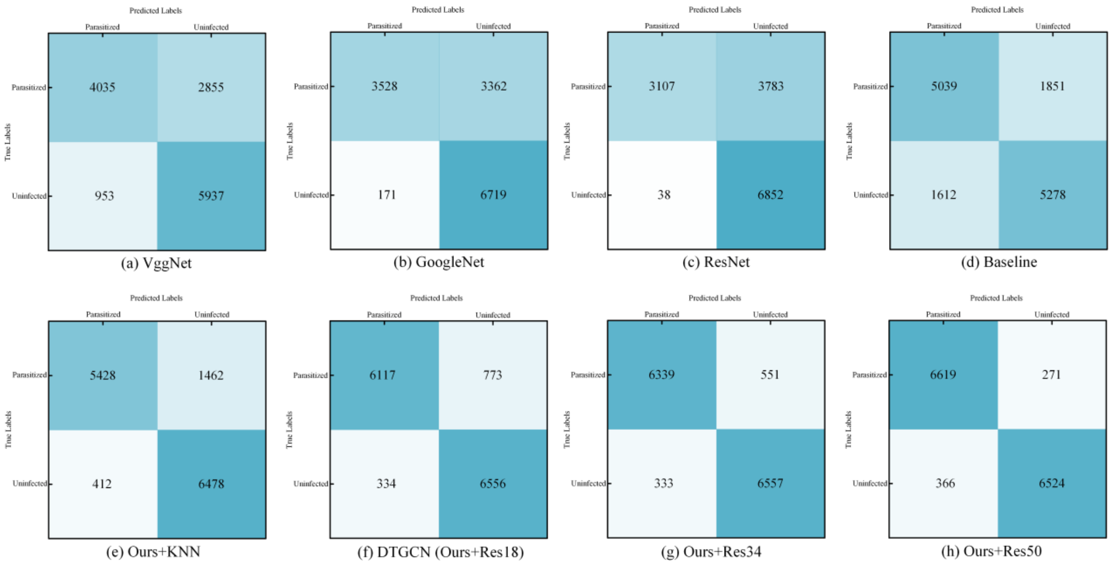


**Figure S3. Confusion matrices for the multi-stage malaria parasites classification.** The confusion matrixes of VggNet (a), GoogLeNet (b), ResNet (c), and Baseline (d) are compared to various DTGCN approaches, including replacing the graph building graph by KNN algorithm (Ours+KNN) (e) , replacing the CNN backbones of ResNet-18 (Ours+Res18) (f), ResNet-34 (Our+Res34) (g), and ResNet-50 (Ours+Res50) (h). Confusion matrix reveals the variation in misclassification between the predicted and true labels. The diagonal cells correspond to samples are correctly classified. The off-diagonal cells correspond to incorrectly classified samples. It is easily to see that Ours+Res50 (DTGCN) presents the best classification results on confusion matrix.

## 7. The impact of source data size for recognition accuracy

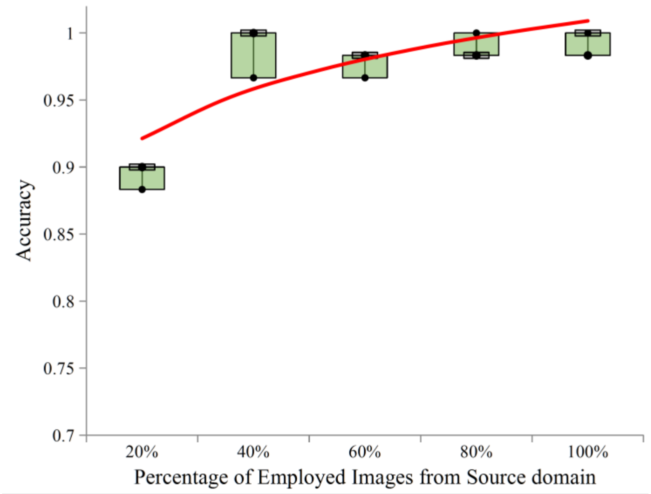


**Figure S4. The impact of source data size for recognition accuracy.** Training the DTGCN model is performed with increasing of the numbers of source examples as reported in a percent (%) of original size (6856). Note that percentage of employed images keeps equivalent ratios of RBC to non-RBC images. For every reported training data size, five repeat trainings are performed. The accuracies are calculated and reported as box plots (n = 5). The results of this study support the fact that a large number of training

images( at least 40%) are necessary for good performance that accuracy is over 90%. This visualization reveals that dataset size plays an important role in achieving high accuracy in classification.

## 8. The visualization of convolutional feature maps

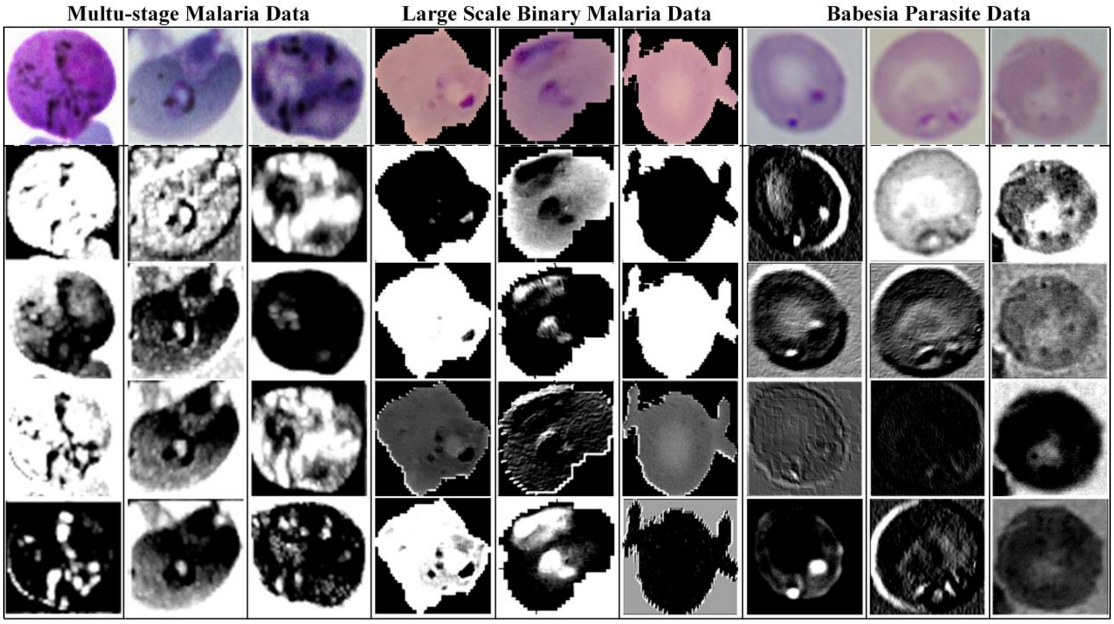


**Figure S5. Visualization of several convolutional feature maps learned by top-3 layers of DTGCN.** To show the evidence of feature detection in more challenging use case examples, we visualize their feature maps from top-3 convolutional layers. Feature map generated from convolutional layers can reveal the detail feature learning procedure in deep learning method. The feature map visualization demonstrates that our DTGCN can extract clear morphological features from these selected challenging images, which can prove that our DTGCN have excellent capability in feature representation for challenging multi-stage malaria parasites recognition.

## References


[1] Maaten, L.v.d. and G.J.J.o.m.l.r. Hinton, *Visualizing data using t-SNE.* 2008. **9**(Nov): p. 2579-2605.
