# Supplementary material for: Multi-stage malaria parasite recognition by deep learning
Source: Gigascience. 2021 Jun 17;10(6):giab040. doi: 10.1093/gigascience/giab040 (PMC8210472; doi:10.1093/gigascience/giab040)
Supplement: giab040_Response_to_Reviewer_Comments_Original_Submission [file giab040_response_to_reviewer_comments_original_submission.pdf]

Author's Response To Reviewer Comments

Close

March 2, 2021

Dear Dr. Nicole,

Thank you for the review of our manuscript entitled “Multi-Stage Malaria Parasites Recognition by Deep Learning" (No. GIGA-D-21-00019). After fully considering the opinions of reviewers and making appropriate and complementary revision (marked in yellow) to the manuscript, we believe it will be of considerable interest to the broad readership of GigaScience. We are hereby resubmitting the revised manuscript, which has been carefully revised according to the reviewers' comments and suggestions. Point-by-point responses to the comments are appended, as well as the revised manuscript.
The authors appreciate your consideration of our manuscript again, and we look forward to hearing from you soon.

With my best regards

Yang Zhang
Email: zhangyang07@hit.edu.cn

Response to reviewers
Response to Reviewer #1
Comments:

(1) Authors have strengthened their paper. However, there are still some existing benchmarks that authors need to consider in their publications as the ones mentioned below for malaria detection:
[1] Narayanan, B. N., Ali, R., & Hardie, R. C. (2019, September). Performance analysis of machine learning and deep learning architectures for malaria detection on cell images. In Applications of Machine Learning (Vol. 11139, p. 111390W). International Society for Optics and Photonics.
[2] Narayanan, B. N., De Silva, M. S., Hardie, R. C., Kueterman, N. K., & Ali, R. (2019). Understanding deep neural network predictions for medical imaging applications. arXiv preprint arXiv:1912.09621.
Response: Many thanks for your comments. The benchmarks [1][2] mentioned above are designed for single-stage malaria parasite recognition. In detail, Narayanan. et al [1] proposed a fast CNN architecture and compared it with AlexNet, ResNet, VGG-16 and DenseNet models for the malaria detection. Findings showed that all tested methods achieved over 96% accuracies. Similar, Narayanan. et al [2] investigated the detection of malaria by using deep neural networks (GoogLeNet and ResNet), and obtained accuracies over than 96%. Following your comments, we add these two works into the revised manuscript and explain them in Section 1 Paragraph 2 (highlighting content).

Response to Reviewer #2
Comments:

(1) Previously the paper only compares the proposed method with baseline models. Now it adds four different methods from other recent papers, ranging from 2016 to 2020. This makes the experimental results more convincing. What can be further improved is adding some comparison and explanation between the competing methods. For example, what methods they use to detect malaria, or what dataset they use on the experiment.
Response: Thank you very much for the comment. As for the baselines of recent papers, Quinn et al [3] designed a deep learning model trained from the annotated cell images with four hidden layers consisting of two convolution layers, one pooling layer and a fully connected layer. The performance of the deep neural networks was evaluated on the detection of malaria parasite in thick blood smears with an average precision of 97%. Rajaraman et al [4] employed pre-trained CNN based deep learning

models as feature extractors to classify parasitized and uninfected cells, obtaining 95.7% classification accuracy on single-stage malaria detection. Vijayalakshmi et al [5] developed a novel transfer learning approach to identify infected malaria parasite, which is powered by combining VggNet and support vector machine. The results on malaria digital corpus images have achieved classification accuracy of 93.1%. Umer et al [6] applied pre-processing steps for re-sampling and normalizing input microscopy images and then utilized stacked CNN by fine-tuning it along with max-pooling and dropout layer. The performance of this model was evaluated on single stage malaria parasite detection, resulting in 99.98% accuracy. These baseline methods are re-trained on the BBBC dataset and tested on the multi-stage malaria parasite images. In the experiments on large scale dataset of malaria parasites recognition, different stages of malaria parasites are all grouped as the infected images. All models trained on the multi-stage malaria parasite images are directly transferred and tested on the large-scale malaria recognition dataset without further training. Compared to DTGCN, recent works are originally proposed for single stage malaria parasite recognition, which is more vulnerable to the variations in multi-stage malaria parasites recognition. The variations degrade the performance of models developed previously, and has only achieved a maximum recognition accuracy of 66.3%. Therefore, simply adaptation of existing single-stage classification models into multi-stage malaria parasites recognition will perform poorly. According to your comments, we have added comparison and explanation on competing methods in Section 3.1, including what methods they use to detect malaria, and what dataset they use on the experiments (highlighting content).

[3]. Quinn J A, Nakasi R, Mugagga P K B, et al. Deep convolutional neural networks for microscopy-based point of care diagnostics[C]//Machine Learning for Healthcare Conference. 2016: 271-281.
[4]. Rajaraman S, Antani S K, Poostchi M, et al. Pre-trained convolutional neural networks as feature extractors toward improved malaria parasite detection in thin blood smear images[J]. PeerJ, 2018, 6: e4568.
[5]. Vijayalakshmi A. Deep learning approach to detect malaria from microscopic images[J]. Multimedia Tools and Applications, 2020, 79(21): 15297-15317.
[6]. Umer M, Sadiq S, Ahmad M, et al. A Novel Stacked CNN for Malarial Parasite Detection in Thin Blood Smear Images[J]. IEEE Access, 2020, 8: 93782-93792.

(2) In Table 2, the proposed DTGCN results are not highlighted in bold as what they are in Table 1.
Response: Very grateful for this comment. According to your comments, we have highlighted the best results in bold in Table 2.

Response to Reviewer #3
Comments:

(1) Do the authors know the pixel resolution of the image data used in this study? If so, can the authors provide these details as an Excel sheet? This will allow me to check these details. If the authors do not know the pixel resolution of the images, can they please comment on why they feel the size of the malaria parasite, for which one would require details of pixel size, is not necessary for an image-based analysis that seeks to determine the stage of malaria parasites?
Response: Thank you very much. We know the detail pixel resolution of the image data (BBBC, Binary Malaria Images, Babesia), and we have summarized them in an Excel sheet, which can be downloaded from dataset (https://data.mendeley.com/datasets/xvs55d4rcz/draft?a=6223e44e-04b8-4705-91d9-bf98665c6194). Please check these details of both malaria parasite datasets and Babesia images. Although these image pixels are different, they have been resized into the same pixel setting by the pre-processing process in the deep learning model. From that, the proposed DTGCN can automatically determine the stage of malaria parasite without considering details of pixel size.

(2) I wish to see illustrative examples of how robustly the DTGCN can handle the different types of variation (staining intensity, illumination) referred to in the manuscript. Figure S1b is highly insightful as it demonstrates the inherent variability in the images used in this study. Furthermore, there is an interesting statement in the manuscript whereby the authors highlight that "the second malaria parasite recognition task in this paper is classification of unseen malaria parasite in a large-scale dataset which has different distributions (such as brightness and imaging equipment settings) from source training dataset." I would like to see evidence of feature detection in these more challenging use case examples.
Response: Many thanks for your comments. To show the evidence of feature detection in more challenging use case examples, we visualize their feature maps from top-3 convolutional layers. Feature map generated from convolutional layers can reveal the detail feature learning procedure in deep learning method, and we choose some challenging malaria parasite images from each stage with different types of variation to show their feature maps in the Figure S5. The feature map visualization

demonstrates that our DTGCN can extract clear morphological features from these selected challenging images, which can prove that our DTGCN have excellent capability in feature representation for challenging multi-stage malaria parasites recognition.

Close
