# Supplementary material for: Multi-stage malaria parasite recognition by deep learning
Source: Gigascience. 2021 Jun 17;10(6):giab040. doi: 10.1093/gigascience/giab040 (PMC8210472; doi:10.1093/gigascience/giab040)
Supplement: giab040_Response_to_Reviewer_Comments_Revision_1 [file giab040_response_to_reviewer_comments_revision_1.pdf]

# Author's Response To Reviewer Comments

Close

Response: Thank you again for the comment. Sorry for our previous misunderstanding of pixel details. We have collected the pixel size details of each image and supplied in the online Excel, which can be download from (https://data.mendeley.com/datasets/xvs55d4rcz/draft?a=6223e44e-04b8-4705-91d9-bf98665c6194). According to the scale bars of the raw images, the average width or height pixel size of our multi-stage malaria images is in 11.842 μm to 18.947 μm.

The deep learning model has excellent feature learning capability by reasoning their representative class information from large amounts of different images with huge variations in size, shape or pixel values [1,2,3]. The pixel values in images must be normalized prior to providing the images as input to a deep learning neural network model during the training or evaluation of the model [4]. In our study, a total of 79672 multi-stage parasitized and uninfected cell images were cropped from raw images according to the given boxing coordinates. Those cell images with different pixel values were resized to 128*128 pixels before feeding into the deep neural networks. That is because each layer of deep neural networks only accepts image inputs of certain pixel value. Therefore, the deep learning models can handle the multi-stage malaria images with different pixel values. Our previous response is misleading, which has been corrected to "As the network accepts image inputs of certain pixel value, the input images with different number of pixels have been resized to 128*128 pixels before feeding into our deep learning model. " According to your comments, we have added this description in Section 2.1 Data Acquisition (highlighting content).

[1] LeCun Y, Bengio Y, Hinton G. Deep learning[J]. nature, 2015, 521(7553): 436-444.
[2] Esteva A, Kuprel B, Novoa R A, et al. Dermatologist-level classification of skin cancer with deep neural networks[J]. nature, 2017, 542(7639): 115-118.
[3] Coudray N, Ocampo P S, Sakellaropoulos T, et al. Classification and mutation prediction from non–small cell lung cancer histopathology images using deep learning[J]. Nature medicine, 2018, 24(10): 1559-1567.
[4] Zheng X, Yao Z, Huang Y, et al. Deep learning radiomics can predict axillary lymph node status in early-stage breast cancer[J]. Nature communications, 2020, 11(1): 1-9.

Close
