# Supplementary material for: Multi-stage malaria parasite recognition by deep learning
Source: Gigascience. 2021 Jun 17;10(6):giab040. doi: 10.1093/gigascience/giab040 (PMC8210472; doi:10.1093/gigascience/giab040)
Supplement: giab040_Response_to_Reviewer_Comments_Revision_2 [file giab040_response_to_reviewer_comments_revision_2.pdf]

Author's Response To Reviewer Comments

Close

Dear Dr. Nicole Nogoy

Thank you for the reviewing of our manuscript. We are hereby resubmitting the revised manuscript, which has been carefully revised according to your comments.

The authors appreciate your consideration of our manuscript again, and we look forward to hearing from you soon.

Sincerely yours

Yang

Close
