# Supplementary material for: Multi-stage malaria parasite recognition by deep learning
Source: Gigascience. 2021 Jun 17;10(6):giab040. doi: 10.1093/gigascience/giab040 (PMC8210472; doi:10.1093/gigascience/giab040)
Supplement: giab040_Reviewer_2_Report_Original_Submission — Michael Rung-Tsong Lyu -- 2/12/2021 Reviewed [file giab040_reviewer_2_report_original_submission.pdf]

Reviewer Report

Title: Multi-Stage Malaria Parasites Recognition by Deep Learning

Version: Original Submission **Date:** 2/12/2021

Reviewer name: Michael Rung-Tsong Lyu

Reviewer Comments to Author:

1. Previously the paper only compares the proposed method with baseline models. Now it adds four different methods from other recent papers, ranging from 2016 to 2020. This makes the experimental results more convincing. What can be further improved is adding some comparison and explanation between the competing methods. For example, what methods they use to detect malaria, or what dataset they use on the experiment.
2. In Table 2, the proposed DTGCN results are not highlighted in bold as what they are in Table 1.
3. The paper adds more details to the introduction of proposed methods compared with the previous version, making it easier to read and eliminating some ambiguity. For example, the one in page 4, Section 2.5 illustrates more clearly how the pipeline between GCN and K-means works.
4. This revised version also addresses my precious concern that how to set the hyper-parameter K in K-means methods.
5. Finally, the paper has fixed many typos mentioned before.

Methods

Are the methods appropriate to the aims of the study, are they well described, and are necessary controls included? Choose an item.

Conclusions

Are the conclusions adequately supported by the data shown? Choose an item.

Reporting Standards

Does the manuscript adhere to the journal's guidelines on [minimum standards of reporting?]() Choose an item.

Choose an item.

Statistics

Are you able to assess all statistics in the manuscript, including the appropriateness of statistical tests used? Choose an item.

Quality of Written English

Please indicate the quality of language in the manuscript: Choose an item.

Declaration of Competing Interests

Please complete a declaration of competing interests, considering the following questions:

- Have you in the past five years received reimbursements, fees, funding, or salary from an organisation that may in any way gain or lose financially from the publication of this manuscript, either now or in the future?
- Do you hold any stocks or shares in an organisation that may in any way gain or lose financially from the publication of this manuscript, either now or in the future?
- Do you hold or are you currently applying for any patents relating to the content of the manuscript?
- Have you received reimbursements, fees, funding, or salary from an organization that holds or has applied for patents relating to the content of the manuscript?
- Do you have any other financial competing interests?
- Do you have any non-financial competing interests in relation to this paper?

If you can answer no to all of the above, write 'I declare that I have no competing interests' below. If your reply is yes to any, please give details below.

I declare that I have no competing interests

I agree to the open peer review policy of the journal. I understand that my name will be included on my report to the authors and, if the manuscript is accepted for publication, my named report including any attachments I upload will be posted on the website along with the authors' responses. I agree for my report to be made available under an Open Access Creative Commons CC-BY license (http://creativecommons.org/licenses/by/4.0/). I understand that any comments which I do not wish to be included in my named report can be included as confidential comments to the editors, which will not be published.

Choose an item.

To further support our reviewers, we have joined with Publons, where you can gain additional credit to further highlight your hard work (see: https://publons.com/journal/530/gigascience). On publication of this paper, your review will be automatically added to Publons, you can then choose whether or not to claim your Publons credit. I understand this statement.

Yes Choose an item.
