# Supplementary material for: Multi-stage malaria parasite recognition by deep learning
Source: Gigascience. 2021 Jun 17;10(6):giab040. doi: 10.1093/gigascience/giab040 (PMC8210472; doi:10.1093/gigascience/giab040)
Supplement: giab040_Reviewer_3_Report_Original_Submission — Chris Armit -- 2/15/2021 Reviewed [file giab040_reviewer_3_report_original_submission.pdf]

Reviewer Report

Title: Multi-Stage Malaria Parasites Recognition by Deep Learning

Version: Original Submission **Date:** 2/15/2021

Reviewer name: Chris Armit

Reviewer Comments to Author:

This Research Article has been re-submitted to GigaScience. As before, the manuscript describes a novel deep learning approach for classifying microscopy images of multi-stage malaria parasites and utilises a Deep Transfer Graph Convolutional Network (DTGCN). In my initial review, my concerns were that the Ground Truth image data of blood smears were manually captured and therefore highly variable in terms of intensity of Giemsa-staining. Furthermore, there was neither scalebar on the blood smear images, nor any details of pixel resolution, and the multi-stage parasitised cell images were made available in a JPEG format, which is a non-standard lossy format that is contraindicated for automated image analysis.

For these reasons, I invited the authors to clarify: 1) how their automated image analysis method dealt with the high variation in staining intensity; 2) whether details of image resolution are entirely unnecessary for the Deep Transfer Graph Convolutional Network classification approach outlined in the manuscript, and; 3) whether there is any merit in using a more conventional lossless image format (TIFF format or similar) as the Ground Truth data for a DTGCN Deep Learning classification similar to that outlined in the manuscript. In addition, I invited the authors to clarify whether the percentage of employed images shown in Figure S4 (i.e. 100%, 80%, 60%, 40%, 20%) represent equivalent ratios of RBC to non-RBC images as this would help us understand whether there is an inherent bias in this ablation study on the impact of source data size.

In the revised manuscript, the authors have now provided some details of image resolution - a range of 100 to 160 pixels in either the x or y dimension - and they have also explained that a key advantage of their CNN network is its ability to deal with variation in illumination, background, and staining intensity. Furthermore, the authors have highlighted that equivalent ratios of RBC to non-RBC images were used in the ablation study. I thank the authors for providing these details. However, the authors have not adequately addressed my questions and I am still a little bit confused by the findings of this manuscript. Consequently, I invite the authors to address the following points.

1. Do the authors know the pixel resolution of the image data used in this study? If so, can the authors provide these details as an Excel sheet? This will allow me to check these details.

If the authors do not know the pixel resolution of the images, can they please comment on why they feel the size of the malaria parasite, for which one would require details of pixel size, is not necessary for an image-based analysis that seeks to determine the stage of malaria parasites?

2. I wish to see illustrative examples of how robustly the DTGCN can handle the different types of variation (staining intensity, illumination) referred to in the manuscript. Figure S1b is highly insightful as it demonstrates the inherent variability in the images used in this study. Furthermore, there is an interesting statement in the manuscript whereby the authors highlight that "the second malaria parasite

recognition task in this paper is classification of unseen malaria parasite in a large-scale dataset which has different distributions (such as brightness and imaging equipment settings) from source training dataset." I would like to see evidence of feature detection in these more challenging use case examples.

Methods

Are the methods appropriate to the aims of the study, are they well described, and are necessary controls included? Choose an item.

Conclusions

Are the conclusions adequately supported by the data shown? Choose an item.

Reporting Standards

Does the manuscript adhere to the journal's guidelines on minimum standards of reporting? Choose an item.

Choose an item.

Statistics

Are you able to assess all statistics in the manuscript, including the appropriateness of statistical tests used? Choose an item.

Quality of Written English

Please indicate the quality of language in the manuscript: Choose an item.

Declaration of Competing Interests

Please complete a declaration of competing interests, considering the following questions:

- Have you in the past five years received reimbursements, fees, funding, or salary from an organisation that may in any way gain or lose financially from the publication of this manuscript, either now or in the future?
- Do you hold any stocks or shares in an organisation that may in any way gain or lose financially from the publication of this manuscript, either now or in the future?
- Do you hold or are you currently applying for any patents relating to the content of the manuscript?
- Have you received reimbursements, fees, funding, or salary from an organization that holds or has applied for patents relating to the content of the manuscript?
- Do you have any other financial competing interests?
- Do you have any non-financial competing interests in relation to this paper?

If you can answer no to all of the above, write 'I declare that I have no competing interests' below. If your reply is yes to any, please give details below.

I declare that I have no competing interests.

I agree to the open peer review policy of the journal. I understand that my name will be included on my report to the authors and, if the manuscript is accepted for publication, my named report including any attachments I upload will be posted on the website along with the authors' responses. I agree for my report to be made available under an Open Access Creative Commons CC-BY license (http://creativecommons.org/licenses/by/4.0/). I understand that any comments which I do not wish to be included in my named report can be included as confidential comments to the editors, which will not be published.

Choose an item.

To further support our reviewers, we have joined with Publons, where you can gain additional credit to further highlight your hard work (see: https://publons.com/journal/530/gigascience). On publication of this paper, your review will be automatically added to Publons, you can then choose whether or not to claim your Publons credit. I understand this statement.

Yes Choose an item.
