# Supplementary material for: Multi-stage malaria parasite recognition by deep learning
Source: Gigascience. 2021 Jun 17;10(6):giab040. doi: 10.1093/gigascience/giab040 (PMC8210472; doi:10.1093/gigascience/giab040)
Supplement: giab040_Reviewer_3_Report_Revision_1 — Chris Armit -- 3/4/2021 Reviewed [file giab040_reviewer_3_report_revision_1.pdf]

Reviewer Report

Title: Multi-Stage Malaria Parasites Recognition by Deep Learning

Version: Revision 1 **Date:** 3/4/2021

Reviewer name: Chris Armit

Reviewer Comments to Author:

I thank the authors for attempting to address my questions. Figure S5 is a helpful addition to the manuscript as it highlights the inherent challenge of detecting diagnostic features of multi-stage malaria parasites in a highly diverse image dataset that shows variability in staining intensity and illumination. From inspection of Figure S5, one of the main diagnostic features that the authors use to classify malaria parasites would appear to be the distribution of spots, which the authors claim they can use to stage malaria parasites. I would be more inclined to believe the validity of this approach if the authors were able to provide pixel size details (e.g. 9 micron x 9 micron) that we would enable me to make measurements of the spots in their image dataset. However, once again the authors have not provided the requested pixel size details. The Excel sheet the authors have provided does not provide pixel size details. To illustrate with one example from the Excel sheet, consider image [a1016_81] where the authors provide the following details: pixel width=194, pixel height=93. Presumably, these refer to the number of pixels in an image. However, to calculate the dimensions - or area - of the various spots that are the features used by the authors to determine the stage of the malaria parasites, I would need to know the pixel size. By pixel size, I mean the dimensions - in microns - of one square pixel. As this image dataset is highly variable, I require these details for each image in the imaging dataset. Without these details, measurements cannot be made on these image data, and the resizing argument used by the authors in their response - "Although these image pixels are different, they have been resized into the same pixel setting by the pre-processing process in the deep learning model" - is quite frankly, absurd. For a third time, I invite the authors to provide pixel size details for each of the images used in their analysis. Pixel size details will enable me to make measurements of the features that the authors use in their automated feature detection analysis. By assessing these measurements, I wish to determine whether the computational analysis outlined in the manuscript is valid.

Methods

Are the methods appropriate to the aims of the study, are they well described, and are necessary controls included? Choose an item.

Conclusions

Are the conclusions adequately supported by the data shown? Choose an item.

Reporting Standards

Does the manuscript adhere to the journal's guidelines on minimum standards of reporting? Choose an item.

Choose an item.

Statistics

Are you able to assess all statistics in the manuscript, including the appropriateness of statistical tests used? Choose an item.

Quality of Written English

Please indicate the quality of language in the manuscript: Choose an item.

Declaration of Competing Interests

Please complete a declaration of competing interests, considering the following questions:

- Have you in the past five years received reimbursements, fees, funding, or salary from an organisation that may in any way gain or lose financially from the publication of this manuscript, either now or in the future?
- Do you hold any stocks or shares in an organisation that may in any way gain or lose financially from the publication of this manuscript, either now or in the future?
- Do you hold or are you currently applying for any patents relating to the content of the manuscript?
- Have you received reimbursements, fees, funding, or salary from an organization that holds or has applied for patents relating to the content of the manuscript?
- Do you have any other financial competing interests?
- Do you have any non-financial competing interests in relation to this paper?

If you can answer no to all of the above, write 'I declare that I have no competing interests' below. If your reply is yes to any, please give details below.

I declare that I have no competing interests.

I agree to the open peer review policy of the journal. I understand that my name will be included on my report to the authors and, if the manuscript is accepted for publication, my named report including any attachments I upload will be posted on the website along with the authors' responses. I agree for my report to be made available under an Open Access Creative Commons CC-BY license (http://creativecommons.org/licenses/by/4.0/). I understand that any comments which I do not wish to be included in my named report can be included as confidential comments to the editors, which will not be published.

Choose an item.

To further support our reviewers, we have joined with Publons, where you can gain additional credit to further highlight your hard work (see: https://publons.com/journal/530/gigascience). On publication of this paper, your review will be automatically added to Publons, you can then choose whether or not to claim your Publons credit. I understand this statement.

Yes Choose an item.
